# Functionalization of β‑Alkylcorrole Using Ag(I) Salts

**DOI:** 10.1021/acs.inorgchem.6c02241

**Published:** 2026-07-27

**Authors:** Martina Marsotto, Giuseppe Pomarico, Manuela Stefanelli, Anna Troiani, Frank R. Fronczek, Kevin M. Smith, Roberto Paolesse

**Affiliations:** † Department of Chemical Science and Technologies, University of Rome Tor Vergata, Rome 00133, Italy; ‡ Department of Chemistry, Sapienza—University of Rome, Rome 00185, Italy; § Department of Chemistry and Technology of Drugs, Sapienza—University of Rome, Rome 00185, Italy; ∥ Department of Chemistry, 5779 Louisiana State University, Baton Rouge, Louisiana 70803, United States

## Abstract

To date, the coordination chemistry of β-alkylcorroles is significantly less developed than that of their triaryl analogues. To address this gap, we investigated the seemingly straightforward synthesis of silver complexes of β-alkylcorroles, employing a procedure previously reported for Ag­(III) insertion into *meso*-triarylcorroles. However, when AgOAc was used as the silver source in pyridine, the reaction resulted in the formation of oxidation products, specifically oxocorroles. In acetonitrile, a broader distribution of products was observed, yielding compounds in addition to oxocorroles. Characterization through UV–vis spectroscopy, ^1^H NMR, mass spectrometry, and single-crystal X-ray crystallography revealed the formation of an acetate-functionalized oxocorrole, along with a second species resulting from simultaneous silver insertion and acetate incorporation at the *meso-*position. Substituting AgOAc with AgNO_2_ demonstrated a marked time dependence: rapid and complete *meso* nitration occurred within minutes, while extended reaction times also facilitated silver insertion into the preformed trinitrocorrole free base. The use of silver salts with non-nucleophilic anions (e.g., AgPF_6_ and AgNO_3_) did not yield either metalation or *meso*-functionalization. These results suggest that the coordination of the silver ion into the corrole core is almost permitted only after peripheral substitution, highlighting the distinct reactivity of alkylcorroles compared to their triaryl counterparts.

## Graphical abstract

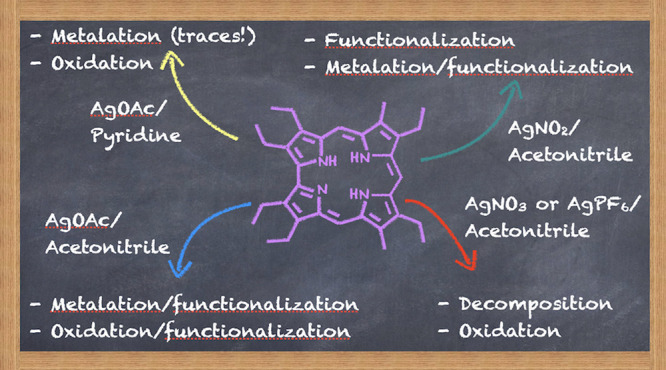

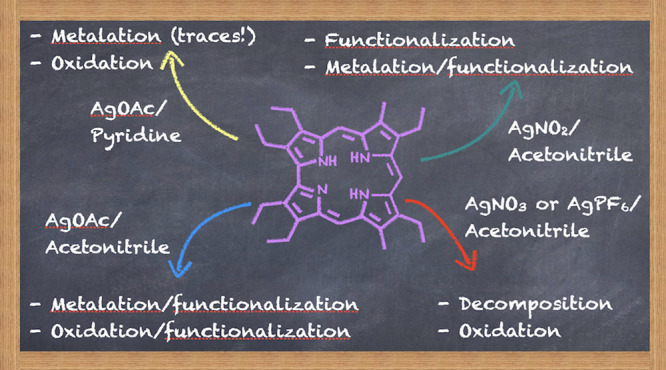

## Introduction

Corroles are tetrapyrrolic macrocycles closely related to porphyrins, but structurally distinct due to the presence of a contracted ring arising from a direct pyrrole–pyrrole bond, while retaining the porphyrin 18 π-electron aromatic system. A key structural feature of this macrocycle is the presence of three NH groups within its inner core, which plays a crucial role in defining its identity as a tetradentate and trianionic ligand. While this latter character enhances its σ-donating ability, facilitating the stabilization of coordinated metal ions in (formally) higher oxidation states, the electron richness makes the macrocycle prone to facile oxidation, which in metal complexes is evidenced by a ligand-to-metal charge transfer process that results in the formation of metal^II^-corrole^•2–^ species, a phenomenon known as ligand noninnocence^1^ and references cited therein. This effect is particularly evident in coinage metal corrole complexes:^2,3^ while gold corroles exhibit an innocent character of the contracted macrocycle ligand, copper derivatives are better described as Cu­(II)-corrole^•2–^ species.^4,5^ Silver, instead, displays a nuanced behavior:^2^ unhindered arylcorrole can be rationalized as innocent Ag­(III)-corrole^3–^ systems, whereas fully substituted β-octabromo derivatives are substantially noninnocent. These challenges in determining the electronic structure of corrole complexes provide valuable opportunities for further chemical investigation and functional manipulation.

The development of efficient synthetic protocols for the preparation of *meso*-triarylcorroles, now accessible in high yields under mild conditions from commercially available reagents,^6–9^ has allowed extensive investigations of their coordination chemistry and peripheral functionalization at the β-pyrrole positions.^10–12^ Functionalization of the molecular scaffold or varying the coordinated metal allows fine-tuning of redox, photophysical, and catalytic properties, making corroles valuable platforms for applications in molecular electronics, catalysis, sensing, and medicine.^13–16^

In contrast, the chemistry of β-alkylcorroles is much less explored, even though they were the first corroles reported by Johnson and Kay in 1965.^17^ The more laborious and demanding synthetic procedures required for their preparation have severely limited the exploration of their chemistry, notwithstanding the significant role that alkyl substituents at the β-pyrrole positions can play in modulating the solubility, conformational rigidity, and electronic distribution within the macrocycle. Compared to *meso*-triarylcorroles, these structural differences introduce additional challenges in both metalation and functionalization reactions.

In comparison with *meso*-substitution exploited in β-alkyl porphyrins,^18^ the analogous reactivity in β-alkylcorroles remains largely uncharted. Only a few examples have been reported in the literature,^19^ and thorough investigations into their reactivity and synthetic potential remain essential. Given that specific functionalization reactions require harsh conditions, metal-ion coordination within the macrocycle is often crucial for protecting it from decomposition or for controlling the regiochemical outcome of the transformation. The simultaneous metalation and *meso*-functionalization of β-alkylcorrole derivatives therefore represents a largely unexplored route to developing novel complexes with tailored structural and electronic properties.

Of the various potential metal derivatives of β-alkylcorroles, the coordination of coinage metal ions can be of particular interest for investigating the influence of the peripheral substitution on the noninnocent character of the corrole ligand. While copper derivatives were among the first corrole complexes synthesized,^17,20^ to the best of our knowledge, only one example of a fully substituted silver complex has been reported in the literature.^21^ Silver ion coordination to 5,10,15-triarylcorroles proceeds in high yield, affording diamagnetic complexes that can be readily characterized by NMR spectroscopy; moreover, the ability of silver to direct the regioselectivity of bromination and nitration reactions, frequently resulting in a single regioisomer,^22,23^ facilitates the synthesis of functionalized corroles that can be further transformed into novel metal complexes by Ag reductive demetalation.^24^ These complexes could exhibit unique electronic or catalytic behavior dictated by the newly introduced metal ion. Unexpectedly, Ag complexation proved more challenging than expected due to the high susceptibility of the macrocycle to oxidation and the remarkable reactivity of its unsubstituted *meso*-positions, ultimately preventing the formation of the desired complex.

Despite this, systematic modulation of the experimental conditions – both the choice of solvent and the nature of the silver salt – provided valuable insights into the reactivity of β-alkylcorroles, with the characterization of novel porphyrinoid derivatives. The results presented here not only deepen the understanding of the coordination chemistry of this underexplored class of corroles but also offer new perspectives on the development of tetrapyrrolic macrocycles with unique structural and functional properties, potentially relevant to various research applications.

## Experimental Section

### General Methods

Reagents and solvents (Aldrich, Merck, Carlo Erba, and TCI) were of the highest grade available and used without further purification or drying prior the use. All reactions were carried out in air (not under an inert atmosphere), and without shielding the reaction flask from light. However, it is essential to store the silver salts protected from light to prevent their decomposition, which in some cases can be observed via a change in color. Thin-layer chromatography (TLC) was performed on Sigma-Aldrich silica gel plates. Silica gel 60 (70–230 mesh, Sigma-Aldrich) or neutral alumina (Brockmann grade III) was used for chromatographic purification. UV–vis spectra were measured on a Varian Cary 60 spectrophotometer using CH_2_Cl_2_ as solvent. ^1^H NMR experiments were performed in deuterated chloroform at 25 °C and recorded with a Bruker Avance spectrometer operating at 700.09 MHz (for ^1^H), equipped with a 5 mm inverse TXI probe and *z*-axis gradients. Chemical shifts are reported in ppm (δ) relative to residual solvent (CHCl_3_: δ = 7.26 ppm). NMR spectra were processed with iNMR or TopSpin 5.0.0. Electrospray Ionization/Quadrupole time-of-flight (ESI/Q-TOF) high-resolution mass spectrometry (HRMS) spectra were recorded on an Agilent 6520 Q-TOF mass spectrometer, using acetonitrile/formic acid (99:1) as solvent.

Accurate mass measurements were carried out on an Orbitrap Exploris 120 mass spectrometer (Thermo Fisher Scientific) equipped with an atmospheric pressure matrix-assisted laser desorption/ionization (AP-MALDI) ion source (MassTech Inc.). Full details of the instrumental setup are described elsewhere.^25^ Synthesized compounds were cocrystallized with α-cyano-4-hydroxycinnamic acid (CHCA, positive ion mode) or 1,2-diaminonaphthalene (DAN, positive- and negative-ion mode) matrices on a MALDI target plate. Spectra were acquired over *m*/*z* 400–1000 at a resolving power of 120000. Compounds were detected as [M + H]^+^, [M – H]^−^, or [M]^+•^/[M]^−•^ ions as appropriate. Full experimental details and spectra are provided in the Supporting Information. Mass accuracy was below 2 ppm in all cases.

The X-ray single-crystal structures of [(AcO)­EMCorr]­Ag, 15-AcO-5-OxoCorrH_2_, and [(NO_2_)_3_EMCorr]Ag were determined using intensity data collected at 100 K with Cu Kα radiation on a Bruker D8 Venture DUO diffractometer equipped with a Photon III C14 detector. 15-AcO-5-OxoCorrH_2_ had three independent molecules in the asymmetric unit, and [(NO_2_)_3_EMCorr]Ag had two. CIFs have been deposited with the Cambridge Crystallographic Data Centre as CCDC 2549278–2549280.

Note: No uncommon, new, or unexpected hazards were identified in this work. Risks were managed by using small volumes or amounts of flammable, hazardous, or oxidizing substances.

### Synthesis

#### 2,3,8,12,17,18-Hexaethyl-7,13-dimethylcorrole [EMCorrH_3_]

EMCorrH_3_ was prepared as previously reported.^26^

#### General Procedure for the Reaction with Silver­(I) Acetate

##### In Pyridine

EMCorrH_3_ (42 mg, 0.085 mmol) was dissolved in 10 mL of pyridine, and AgOAc (47 mg, 0.282 mmol) was added. The resulting solution was heated at 50 °C, and the progress of the reaction was monitored by TLC and UV–vis spectroscopy. In 1 h, the starting material was completely consumed. The reaction mixture was then filtered through a Celite plug, and the solvent was removed under reduced pressure. Finally, the residue was subjected to chromatography on silica gel, eluting with CH_2_Cl_2_/hexane (7:3 v/v). Three main fractions were isolated: the first pink fraction corresponded to traces of the silver complex [EMCorr]­Ag. The other two fractions were crystallized as brown-orange powders by using a CH_2_Cl_2_/hexane mixture and were identified as the 10-oxocorrole (10-OxoCorrH_2_, 8% yield) and the 5-oxocorrole (5-OxoCorrH_2_, 18% yield) derivatives by a comparison with similar oxocorroles reported in the literature.^27^

*2,3,8,12,17,18-Hexaethyl-7,13-dimethyl-10-oxocorrole (10-OxoCorrH_2_):* ^1^H NMR (700 MHz, CDCl_3_) δ, ppm: 20.64 (s, 2H, −NH), 5.44 (s, 2H, 5,15-*meso*), 2.42 (q, 4H, *J* = 7.41 Hz, −C*H*_2_CH_3_), 2.12 (q, 4H, *J* = 7.49 Hz, −C*H*_2_CH_3_), 2.10 (q, 4H, *J* = 7.63 Hz, −C*H*_2_CH_3_), 1.66 (s, 6H, −CH_3_), 1.00–0.96 (overlapping t, 12H, −CH_2_C*H*_3_), 0.91 (t, 6H, *J* = 7.42 Hz, −CH_2_C*H*_3_). ESI/Q-TOF (*m/z*) calcd for C_33_H_41_N_4_O [M + H]^+^ 509.3280, found 509.3465. UV–vis (CH_2_Cl_2_), λ_max_ [nm] ε [M^–1^ cm^–1^]: 318 (5.94 × 10^3^), 409 (1.04 × 10^4^).

*2,3,8,12,17,18-Hexaethyl-7,13-dimethyl-5-oxocorrole (5-OxoCorrH_2_):* ^1^H NMR (700 MHz, CDCl_3_) δ, ppm: 22.14 (s, 1H, −NH), 21.35 (s, 1H, −NH), 5.39 (s, 1H, *meso*), 5.03 (s, 1H, *meso*), 2.39 (q, 2H, *J* = 7.42 Hz, −C*H*_2_CH_3_), 2.16 (q, 2H, *J* = 7.56 Hz, −C*H*_2_CH_3_), 2.11 (q, 2H, *J* = 7.49 Hz, −C*H*_2_CH_3_), 2.08–2.02 (overlapping q, 6H, −C*H*_2_CH_3_), 2.00 (q, 2H, *J* = 7.56 Hz, −C*H*_2_CH_3_), 1.96 (s, 3H, −CH_3_), 1.57 (s, 3H, −CH_3_), 1.00 (t, 3H, *J* = 7.42 Hz, −CH_2_C*H*_3_), 0.98–0.94 (overlapping t, 9H, −CH_2_C*H*_3_), 0.92 (t, 3H, *J* = 7.56 Hz, −CH_2_C*H*_3_), 0.89 (t, 3H, *J* = 7.63 Hz, −CH_2_C*H*_3_). ESI/Q-TOF (*m/z*) calcd for C_33_H_41_N_4_O [M + H]^+^ 509.3280, found 509.3178. UV–vis (CH_2_Cl_2_), λ_max_ [nm] ε [M^–1^ cm^–1^]: 327 (2.62 × 10^4^), 371 (3.27 × 10^4^).

##### In Acetonitrile

EMCorrH_3_ (42 mg, 0.085 mmol) was dissolved in 20 mL of CH_3_CN, and AgOAc (47 mg, 0.282 mmol) was added. The resulting solution was heated at 50 °C, and the progress of the reaction was monitored by TLC and UV–vis spectroscopy. The color of the solution changed from purple to deep purple as the reaction proceeded. Upon completion (1.5 h), the reaction mixture was filtered through a Celite plug, the solvent was removed under reduced pressure, and the residue was chromatographed on silica gel. The column was eluted with CH_2_Cl_2_/hexane (7:3 v/v) to afford five main fractions in the following order: the first fraction corresponded to 10-OxoCorrH_2_ (5% yield), the second magenta fraction corresponded to the [(AcO)­EMCorr]Ag (6% yield), the third fraction corresponded to 5-OxoCorrH_2_ (9% yield), the fourth brown fraction corresponded to 15-Aco-5-OxoCorrH_2_ (11% yield), and the last magenta fraction to the [(AcO)_2_EMCorr]Ag (traces).

*[5-Acetoxy-2,3,8,12,17,18-hexaethyl-7,13-dimethylcorrolato]­silver­(III) ([(AcO)­EMCorr]­Ag):* ^1^H NMR (700 MHz, CDCl_3_) δ, ppm: 9.52 (s, 1H, 15-*meso*), 9.39 (s, 1H, 10-*meso*), 4.12–4.05 (m, 4H, −C*H*_2_CH_3_), 4.00–3.88 (m, 7H, −C*H*_2_CH_3_), 3.74–3.69 (m, 1H, −C*H*_2_CH_3_), 3.49 (s, 3H, −CH_3_), 3.46 (s, 3H, −CH_3_), 2.94 (s, 3H, −O­(O)­CCH_3_), 1.88 (t, 3H, *J* = 7.78 Hz, −CH_2_C*H*_3_) 1.85–1.82 (m, 9H, −CH_2_C*H*_3_), 1.79–1.76 (overlapping t, 6H, −CH_2_C*H*_3_). HRMS (AP-MALDI): monoisotopic *m*/*z* calcd for C_35_H_41_^107^AgN_4_O_2_ [M]^+•^ 656.2275, found 656.2267. UV–vis (CH_2_Cl_2_), λ_max_ [nm] (ε, M^–1^ cm^–1^): 410 (6.25 × 10^4^); 486 (2.14 × 10^3^); 521 (4.36 × 10^3^); 557 (2.05 × 10^4^).

*[5,10-Diacetoxy-2,3,8,12,17,18-hexaethyl-7,13-dimethylcorrolato]­silver­(III) HRMS (AP-MALDI) ([(AcO)_2_EMCorr]­Ag):* Monoisotopic *m/z* calcd for C_37_H_43_^107^AgN_4_O_4_ [M]^+•^ 714.2330, found 714.2326.

*15-Acetoxy-2,3,8,12,17,18-hexaethyl-7,13-dimethyl-5-oxocorrole (15-AcO-5-OxoCorrH_2_):* ^1^H NMR (700 MHz, CDCl_3_) δ, ppm: 21.26 (s, 1H, −NH), 20.57 (s, 1H, −NH), 5.55 (s, 1H, 10-*meso*), 2.44 (q, 2H, *J* = 7.42 Hz, −C*H*_2_CH_3_), 2.29–2.24 (m, 1H, −C*H*_2_CH_3_), 2.23–2.21 (m, 2H, −C*H*_2_CH_3_), 2.20 (s, 3H, −O­(O)­CCH_3_), 2.16 (q, 2H, *J* = 7.56 Hz, −C*H*_2_CH_3_), 2.14–2.09 (m, 2H, −C*H*_2_CH_3_), 2.06 (q, 2H, *J* = 7.63 Hz, −C*H*_2_CH_3_), 2.00 (s, 3H, −CH_3_), 1.94–1.88 (m, 1H, −C*H*_2_CH_3_), 1.69 (s, 3H, CH_3_), 1.02 (t, 3H, *J* = 7.42 Hz, −CH_2_C*H*_3_), 0.99–0.94 (m, 9H −CH_2_C*H*_3_), 0.93–0.90 (overlapping t, 6H, *J* = 7.42 Hz, −CH_2_C*H*_3_). HRMS (AP-MALDI): monoisotopic *m/z* calcd for C_35_H_43_N_4_O_3_ [M + H]^+^ 567.3330, found 567.3325. UV–vis (CH_2_Cl_2_), λ_max_ [nm] ε [M^–1^ cm^–1^]: 327 (3.51 × 10^4^); 361 (3.96 × 10^4^).

#### General Procedure for the Reaction with Ag­(I) Nitrite

EMCorrH_3_ (41 mg, 0.083 mmol) was dissolved in 20 mL of CH_3_CN, then NaNO_2_ (57 mg, 0.830 mmol) and AgNO_2_ (64 mg, 0.410 mmol) were added. The mixture was stirred at 50 °C, and the progress of the reaction was monitored by TLC and UV–vis spectroscopy. The color of the solution changed from purple to brownish as the reaction proceeded. Upon completion (30 min), the reaction mixture was filtered through a Celite plug, the solvent was removed on a rotary evaporator, and the residue was chromatographed on silica gel. The column was eluted with CH_2_Cl_2_/hexane (7:3 v/v) to afford a reddish band eluted first, followed by traces of 10- and 5-Oxocorrole species. The first band was crystallized from CH_2_Cl_2_/CH_3_OH to give reddish crystals corresponding to [(NO_2_)_3_EMCorr]Ag (13% yield). When the reaction was performed at room temperature, the solution turned rapidly from purple to green, showing complete consumption of the corrole in 10 min. The same reaction workup as previously described afforded by chromatographic purification the free base (NO_2_)_3_EMCorrH_3_ as a green fraction, which was crystallized from CH_2_Cl_2_/CH_3_OH to give the nitrocorrole as a dark green powder (20% yield).

##### [5,10,15-Trinitro-2,3,8,12,17,18-hexaethyl-7,13-di­methyl­corrolato]­silver­(III) [(NO_2_)_3_­EMCorr]­Ag

^1^H NMR (700 MHz, CDCl_3_) δ, ppm: 4.02 (q, 4H, *J* = 7.77 Hz, −C*H*_2_CH_3_), 3.64 (q, 4H, *J* = 7.70 Hz, −C*H*_2_CH_3_), 3.54 (q, 4H, *J* = 7.70 Hz, −C*H*_2_CH_3_), 3.23 (s, 6H, −CH_3_), 1.79 (t, 6H, *J* = 7.77 Hz, −CH_2_C*H*_3_), 1.66 (t, 6H, *J* = 7.70 Hz, −CH_2_C*H*_3_), 1.56 (t, 6H, *J* = 7.70 Hz, −CH_2_C*H*_3_). HRMS (AP-MALDI): monoisotopic *m/z* calcd for C_33_H_36_^107^AgN_7_O_6_ [M]^−•^ 733.1784, found 733.1786. UV–vis (CH_2_Cl_2_), λ_max_ [nm] (ε, M^–1^ cm^–1^): 417 (5.39 × 10^4^); 533 (9.03 × 10^3^); 567 (1.73 × 10^4^).

##### 5,10,15-Trinitro-2,3,8,12,17,18-hexaethyl-7,13-dimethylcorrole ((NO_2_)_3_EMCorr)

^1^H NMR (700 MHz, CDCl_3_) δ, ppm: 3.85 (q, 4H, *J* = 7.70 Hz, −C*H*_2_CH_3_), 3.46 (q, 4H, *J* = 7.63 Hz, −C*H*_2_CH_3_), 3.29 (q, 4H, *J* = 7.56 Hz, −C*H*_2_CH_3_), 3.01 (s, 6H, −CH_3_), 1.60 (t, 6H, *J* = 7.63 Hz, *–*CH_2_C*H*_3_), 1.44 (t, 6H, *J* = 7.63 Hz, *–*CH_2_C*H*_3_), 1.39 (t, 6H, *J* = 7.70 Hz, *-*CH_2_C*H*_3_). HRMS (AP-MALDI): monoisotopic *m/z* calcd for C_33_H_40_N_7_O_6_ [M + H]^+^ 630.3035, found 630.3033 and *m/z* calcd for C_33_H_38_N_7_O_6_ [M – H]^−^ 628.2889, found 628.2894. UV–vis (CH_2_Cl_2_), UV–vis (CH_2_Cl_2_), λ_max_ [nm] (ε, M^–1^ cm^–1^): 366 (4.03 × 10^4^); 451 (5.74 × 10^4^); 610 (1.91 × 10^4^).

## Results and Discussion

Silver complexes of triarylcorroles in 60–80% yields were first reported by Brückner and co-workers.^28^ The metalation reaction proceeds via Ag­(I) disproportionation, upon reaction of the corresponding triarylcorroles with 3.3 equiv of AgOAc in pyridine at 80 °C. Notably, the metal insertion was found to be highly dependent on the amount of silver­(I) acetate used, as its reduction decreased the reaction yield. In contrast, the reaction remained unaffected by atmospheric O_2_.

The use of similar reaction conditions provided a logical foundation for our investigations, as summarized in Scheme 1. EMCorrH_3_ (Figure S1) was reacted with AgOAc in a (1:3.3) molar ratio in pyridine. After 1 h, TLC analysis indicated complete consumption of the starting material and the formation of three main fractions accompanied by polar decomposition products.

**Scheme 1:**
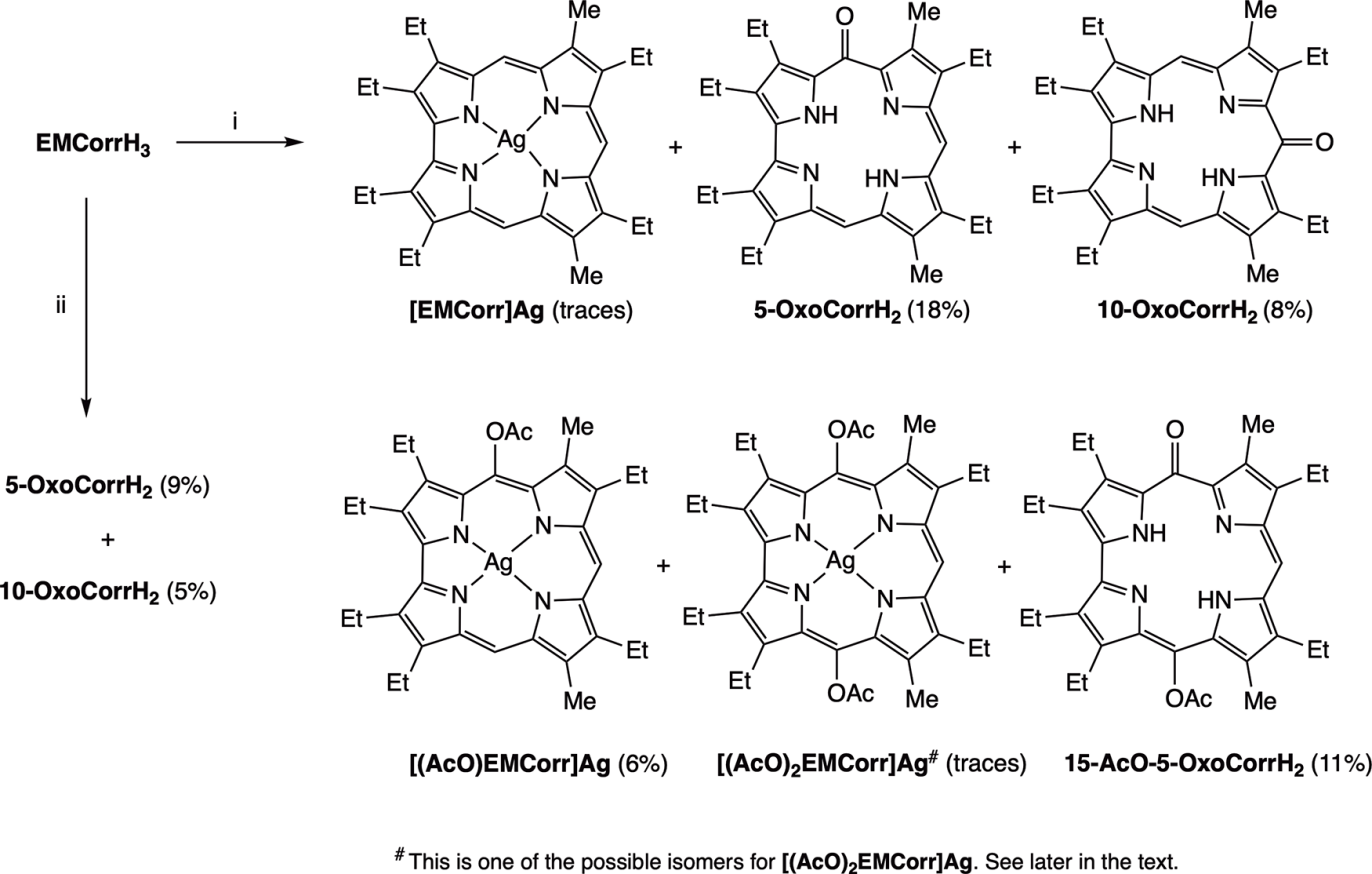
Products of the Reaction of EMCorrH_3_ with 3.3 equiv of AgOAc: (i) Pyridine, 50 °C, 1 h; (ii) Acetonitrile, 50 °C, 1.5 h

Subsequent chromatographic purification yielded a first fraction characterized by a pink hue, the UV–vis spectrum of which closely resembled that of a previously reported silver triarylcorrole.^23,28^ The UV–vis absorption spectrum displayed a Soret band at 409 nm and a prominent Q-band at 559 nm, along with several weaker absorptions that were blue-shifted relative to the Q-band (Figure S2, purple line). Although this product was obtained in a limited amount, thus preventing its complete characterization, UV–vis spectroscopy and AP-MALDI mass spectrometry strongly support the formation of [EMCorr]­Ag. The AP-MALDI mass spectrum in positive ion mode of the isolated fraction displayed radical cation signals at *m*/*z* 598.2218 and 600.2212, in excellent agreement with the calculated values for [C_33_H_39_^107^AgN_4_]^+•^ (calcd 598.2220) and [C_33_H_39_^109^AgN_4_]^+•^ (calcd 600.2217), respectively, and exhibiting the characteristic isotope pattern of a monosilver species, thereby confirming the formation of the silver alkylcorrole [EMCorr]Ag (C_33_H_39_AgN_4,_ Figure S3).

The other two brownish bands corresponded to the primary reaction products. The UV–vis spectra of these fractions (Figure S4) show a Soret-like band in the blue region, with the peak of the 10-isomer red-shifted by almost 40 nm compared to the 5-isomer (409 vs 371 nm) and a very weak, unresolved absorbance above 700 nm. A similar trend has been recently reported for 5-, 10-, and 15-oxoisocorroles obtained via the oxidation of an asymmetric β-alkylcorrole.^29^ These data suggest that the oxidation of *meso*-positions leads to the formation of a 16 π conjugation pattern with a different energy gap among the frontier orbitals involved in the transition, generating the distinct absorbance profiles for the different isomers. Furthermore, the absence of well-defined absorptions above 500 nm (i.e., in the Q-band region) suggests that the macrocyclic conjugation has been partially lost.

Further structural insights were obtained through ^1^H NMR spectroscopy (Figures S5 and S6). The diamagnetic nature of these compounds facilitated the observation of a *meso* proton singlet upfield (5–6 ppm) relative to the starting corrole.

Additionally, resonances above 20 ppm suggested the presence of two residual inner NH protons; such a high chemical shift for inner hydrogens is due to the superposition of two effects, namely, intramolecular H bond and the antiaromatic character of the macrocycle.^30–32^ Overall, the spectroscopic data support the formation of the antiaromatic 5- and 10-oxocorroles. In detail, for the 10-oxocorrole isomer, one singlet integrating for the two inner hydrogens is observed at 20.6 ppm. This behavior suggests a rapid interconversion on the NMR time scale between the two equivalent tautomeric forms (both with hydrogen atoms of the opposite pyrroles), which averages the chemical shifts of the internal protons that would otherwise have different chemical environments. Conversely, in the case of isomer 5, the two possible tautomers are significantly different due to the lower symmetry of the molecule. The observation of two NH signals (1H each) could be attributed to a rapid equilibrium, as for the 10-isomer, or to the strong predominance of a single, thermodynamically favored tautomeric form.

This class of compounds was first observed several years ago during attempts to coordinate Ni­(II) in face-to-face biscorrole and porphyrin–corrole systems.^33^ More recently, they were isolated and structurally characterized by some of us in the course of studies aimed at the preparation of Zn­(II) complexes of β-octaethylcorrole.^27^

The intrinsic propensity of electron-rich β-alkylcorroles to undergo electron abstraction, coupled with the oxidizing nature of Ag­(I), makes macrocycle oxidation highly competitive with the metalation process, resulting in the predominant formation of 5- and 10-oxocorroles, with only trace amounts of the desired Ag complex of the alkylcorrole. This issue does not arise with triarylcorroles, which exhibit greater resistance to oxidation compared to their electron-rich β-alkylated counterparts.^34^ To evaluate whether rapid macrocycle oxidation inhibits metal insertion, we conducted metalation experiments in pyridine using 5-oxocorrole as the substrate and AgOAc as the metal source. Under these conditions, no coordination of the silver ion was observed, suggesting that macrocycle oxidation occurs prior to metal coordination, thereby preventing subsequent formation of the metal complex.

We subsequently focused on acetonitrile as a preferred solvent. This solvent dissolves many silver salts, and the oxidizing power of Ag­(I) may be diminished due to the cation’s stabilization by interactions with four solvent molecules. It has been reported that the solute–solvent interaction does not involve the nitrogen lone pair; instead, acetonitrile acts as a σ-donor, providing electron density from its π orbital to Ag­(I).^35^

With this consideration in mind, we carried out the reaction in acetonitrile and, somewhat unexpectedly, markedly different results compared to those obtained in pyridine were observed. The reaction with 3.3 equiv of AgOAc at 50 °C yielded a more complex mixture of products, as indicated by TLC analysis (silica gel, CH_2_Cl_2_/petroleum ether 7:3, v/v). Two of the bands, corresponding to the first and third fractions, were identified as the previously mentioned 10- and 5-oxocorrole species. Thus, despite selecting acetonitrile to mitigate the salt’s oxidizing power, the macrocycle remained susceptible to oxidation.

The UV–vis spectrum of the second isolated fraction, characterized by a magenta color, closely resembles that obtained from the reaction conducted in pyridine, indicating the formation of a corrole complex. The spectrum features a Soret band centered at 410 nm and two Q-bands at 521 and 557 nm (Figure S2, orange line). The ^1^H NMR spectrum (Figure 1) reveals two distinct singlets at 9.52 and 9.39 ppm, each integrating for one proton corresponding to two hydrogen atoms directly bound to an aromatic corrole. However, the presence of two nonequivalent proton signals indicates the formation of an asymmetric derivative, where one *meso-*position (C-5) has been functionalized. This interpretation is further supported by the appearance of a new singlet at 2.94 ppm, integrating for three protons.

**Figure 1:**
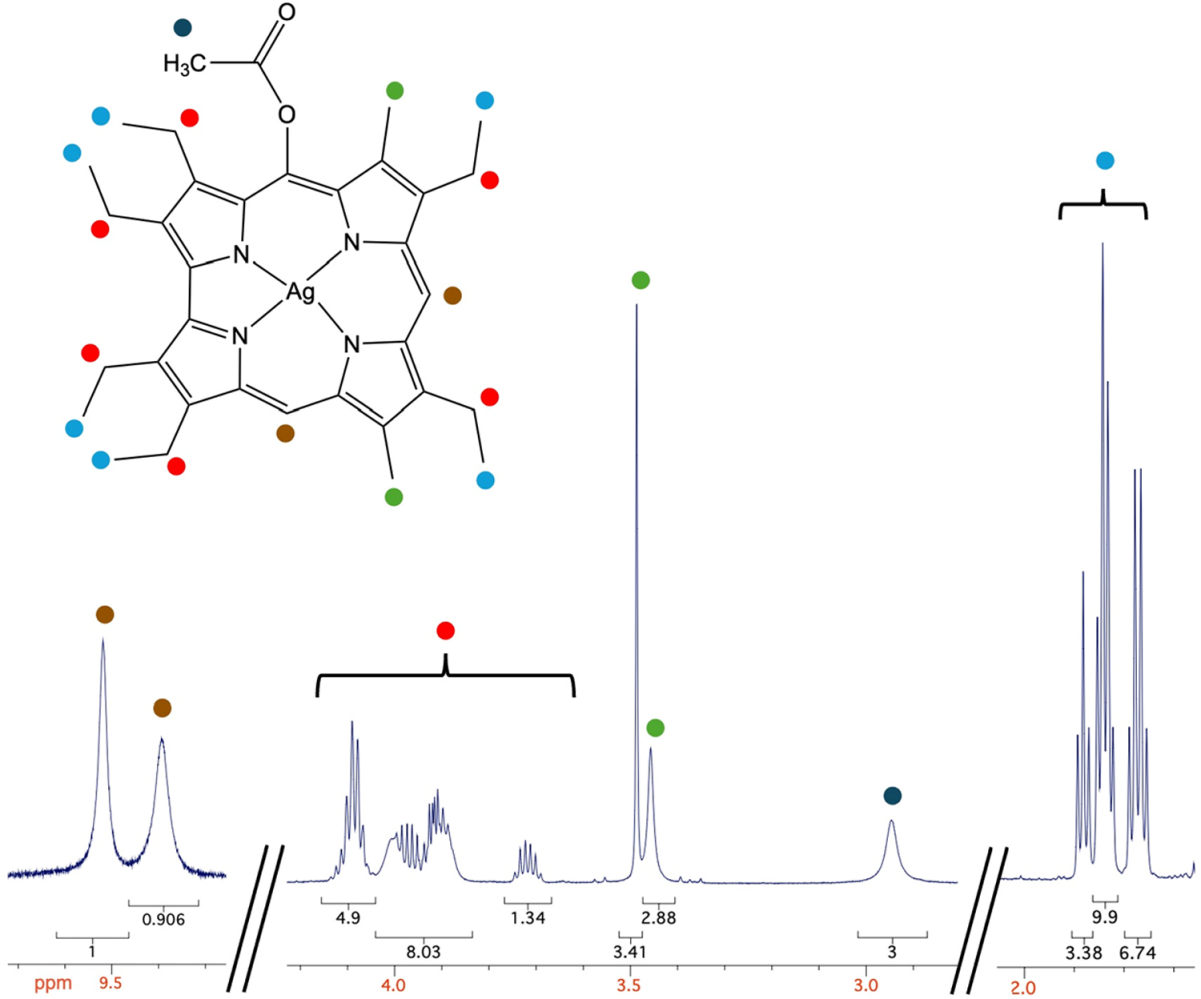
^1^H NMR spectrum of [(AcO)­EMCorr]­Ag. The intensity of signals around 9.5 ppm has been increased for clarity.

These features suggested the insertion of an acetate group at the C-5 *meso*-position of the corrole ring (Figure 1), as confirmed by mass spectrometric analysis.

Consistent with the molecular formula C_35_H_41_AgN_4_O_2_, the AP-MALDI-HRMS spectrum (positive mode) of [(AcO)­EMCorr]Ag showed two radical cation signals at *m*/*z* 656.2267 and 658.2264 (calculated for [C_35_H_41_^107^AgN_4_O_2_]^+•^ 656.2275 and [C_35_H_41_^109^AgN_4_O_2_]^+•^ 658.2272, respectively), displaying the characteristic two-peak isotope pattern expected for a monosilver complex (Figure S9).

Furthermore, the slow diffusion of methanol into a chloroform solution of this compound yielded crystals suitable for single-crystal X-ray crystallographic analysis, enabling an unambiguous identification of the compound as [(AcO)­EMCorr]­Ag. This analysis confirmed the C5 *meso*-position as the site of functionalization (Figure 2).

**Figure 2:**
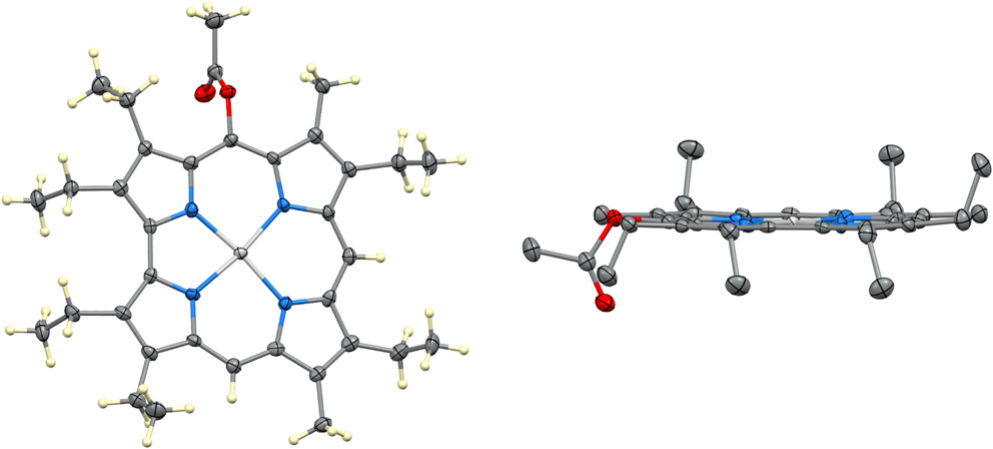
X-ray single-crystal structure of [(AcO)­EMCorr]­Ag, top view and side view. Ellipsoids are at the 50% probability level.

The 23 atoms of the corrole ring system of [(AcO)­EMCorr] are coplanar to within an average deviation of 0.018 Å, and the Ag atom lies only 0.034 Å from this plane. The acetate is nearly perpendicular to the corrole, forming a dihedral angle of 89.5°. The Ag–N distances to the directly linked pyrrole rings are shorter, 1.930(4) and 1.935(4) Å, than the other two, 1.962(4) Å.

The formation of [(AcO)­EMCorr]Ag can follow the same path observed for the β-functionalization of triarylcorroles,^21,36^ with the initial electron abstraction by Ag­(I), leading to the macrocycle radical cation that undergoes nucleophilic attack by the acetate ion. A subsequent one-electron oxidation, accompanied by proton loss, restores the aromaticity of the macrocycle. The electron-withdrawing nature of the acetate group partially offsets the electron-rich character of the macrocycle, thereby enhancing its stability and facilitating metal insertion.

The fourth brownish fraction displayed an absorption spectrum that was quite similar to that of 5-oxocorrole (Figure S4) regarding the wavelength and peak ratio. However, the ^1^H NMR spectrum revealed the presence of only one *meso*-H signal, indicating an additional functionalization of the *meso*-position with respect to that observed for the other derivatives. Furthermore, a singlet integrating for three protons was observed at approximately 2.20 ppm in the ^1^H NMR spectrum, while two resonances (1H each) in the highly deshielded region indicate the formation of an oxocorrole derivative (Figure S10). On the basis of this information, the compound was identified as 15-AcO-5-OxoCorrH_2_, and its structure was subsequently confirmed unambiguously through mass analysis (Figure S11) and single-crystal X-ray crystallographic analysis (Figure 3).

**Figure 3:**
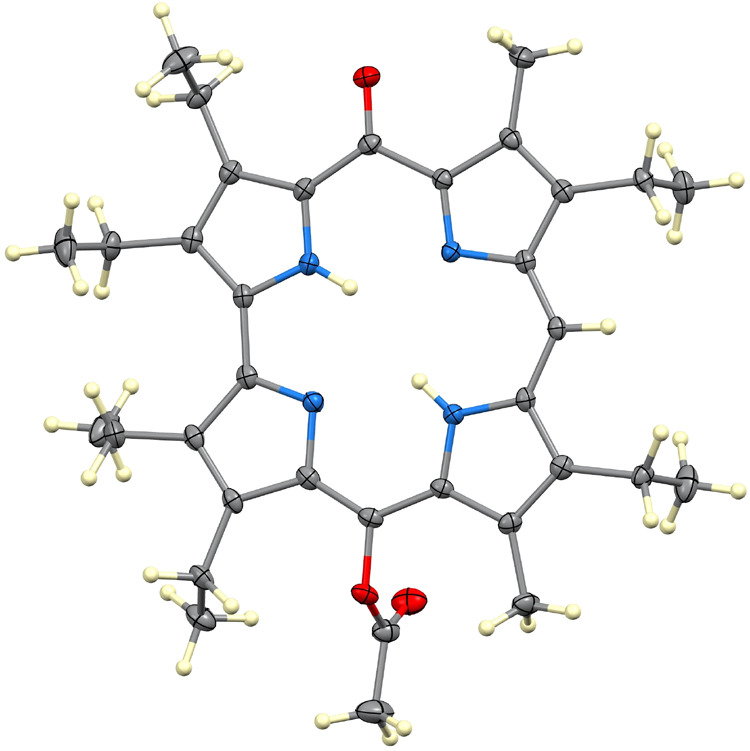
X-ray single-crystal structure of one of the three independent molecules of 15-AcO-5-OxoCorrH_2_, with 50% ellipsoids.

In this derivative, the corrole core of 15-AcO-5-OxoCorrH_2_ is planar to within 0.022 Å (average of 3), and the corrole core forms a dihedral angle of 82.6° (average of 3).

The crystallographic structure we obtained shares several key features with the recently reported structures of 5- and 15-oxocorrole.^29^ In all of these molecular structures, the inner hydrogen adjacent to the C=O moiety is consistently localized on one of the pyrrole rings of the direct bipyrrole unit, indicating that the carbonyl function stabilizes this specific tautomer. If such behavior is retained in solution, it would account for the presence of only two resonances for the core protons, despite the molecule’s asymmetry. Alternatively, the number of observed signals might arise from a rapid tautomeric equilibrium. Additional studies at variable temperatures or 2-D NMR experiments would be necessary to clarify this aspect. Although it is interesting, we have not further investigated it in this work. It is to be underscored that the isolation of a functionalized oxocorrole is particularly noteworthy, as, to the best of our knowledge, this is the first example reported in the literature.

The fifth fraction, the UV–vis spectrum of which closely overlaps with that of [(AcO)­EMCorr]Ag (Figure S2, green line), was identified by mass spectrometry as the difunctionalized complex [(AcO)_2_EMCorr]Ag (Figure S12). Unfortunately, this compound was isolated only in traces, which prevented a complete characterization by NMR and single-crystal X-ray crystallography that would have allowed the position of the second acetate group to be unequivocally identified.

The product distribution observed upon treatment of EMCorrH_3_ with AgOAc in CH_3_CN suggests a multistep pathway, with a first common phase (Scheme 2) being the initial oxidation of the corrole ring by Ag­(I) ion or by the stronger oxidant Ag­(III) formed by Ag­(I) disproportionation in the reaction medium, which leads to a radical cation species. This derivative can then undergo two competitive subsequent reactions: further oxidation to oxocorrole, or nucleophilic attack and subsequent metalation by silver acetate, which generates an acetate-substituted silver complex. The sequential repetition of this process—specifically, the further electronic activation of the macrocycle following the *meso-*position functionalization—accounts for the formation of the difunctionalized *meso*-diacetoxy silver complex.

**Scheme 2:**
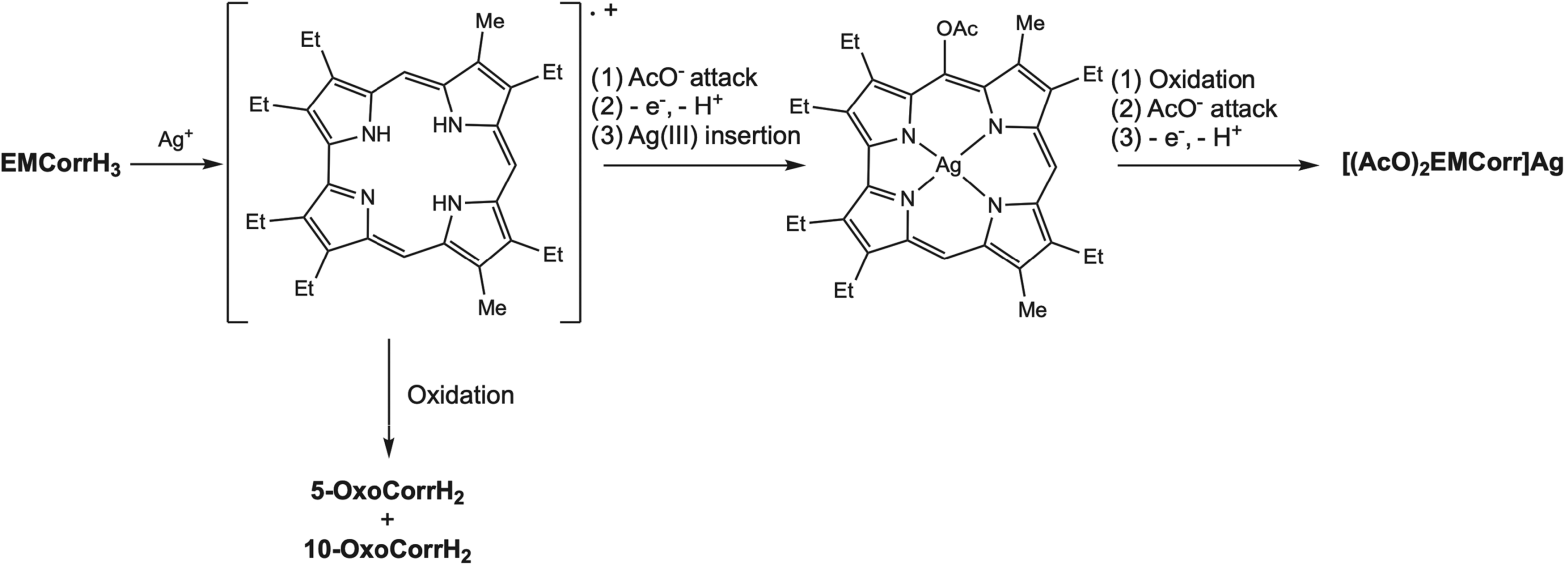
Proposed Mechanism for the Peripheral Functionalization of EMCorrH_3_ by the Reaction with AgOAc in CH_3_CN

Once one *meso*-position is substituted, the macrocycle becomes more susceptible to a second analogous C–H functionalization at the electronically favored 15-position. This reaction pathway is further facilitated by the choice of solvent. While CH_3_CN efficiently stabilizes Ag­(I), it does not establish strong interactions with the acetate.^37^ Consequently, the anion remains poorly solvated and readily attacks the macrocycle, promoting functionalization.

The formation of oxocorrole species can be rationalized as arising from hydrolytic/oxidative conversion of a *meso*-substituted intermediate. A similar oxophlorin derivative has been reported^38^ as a byproduct of the treatment of a β-alkylporphyrin with thallium­(III) trifluoroacetate (a strong oxidant). In this case, the insertion of the trifluoroacetate at the *meso*-position was observed, and the product then underwent hydrolysis to yield the final oxyporphyrin. In one possible scenario, a similar mechanism may explain the formation of oxocorrole, due to the presence of silver ion as the oxidant and the acetate group (or water) acting as nucleophile. Alternatively, oxidation of a *meso*-metalated intermediate to a carbonyl group may occur, with adventitious O_2_ or trace water acting as the formal oxygen source; either pathway ultimately yields the observed 5-oxo-15-acetoxy product. Since acetate insertion is not observed when the metalation reaction is performed directly on oxocorrole, we conclude that macrocycle oxidation prevents further functionalization. In contrast, a macrocycle bearing an acetate substituent can still undergo subsequent oxidation.

The mechanism proposed here is analogous to that observed in our previous studies on nitration reactions.^21,36^ To validate the proposed mechanism and evaluate the effectiveness of this procedure for introducing nitro groups into the alkylcorrole framework, we investigated the metalation/nitration of EMCorrH_3_ by replacing AgOAc with AgNO_2_.

Among the various functionalizations of tetrapyrroles, nitration is particularly attractive, as the nitro group serves as a versatile handle for subsequent transformations.^39^ The nitro substituent significantly influences the optical and electrochemical properties of the chromophore,^40–42^ and its reduction to an amino group introduces a functional moiety with push–pull characteristics that are opposite to those of the nitro group. Furthermore, once reduced to the corresponding amine, it can be used to attach additional units via stable amide bonds.^43^

A wide variety of nitrating systems have been successfully employed for the preparation of β-nitro derivatives of tetraphenylporphyrins.^39^ In the case of triarylcorroles, several systems have also been investigated, yielding various β-nitrocorrole derivatives, both as free bases and as metal complexes.^10,21,43–47^

Considering previous results reported for the nitration of triarylcorroles,^21^ EMCorrH_3_ was reacted with the AgNO_2_/NaNO_2_ system. We found that when an optimized (1:5:10) molar ratio of reactants was used at 50 °C, partial oxidation of the starting material was observed; however, the formation of different products could be controlled by simply adjusting the reaction time (Scheme 3). After 30 min, the reaction produced a reddish fraction in good yield, along with the oxocorrole species.

**Scheme 3:**
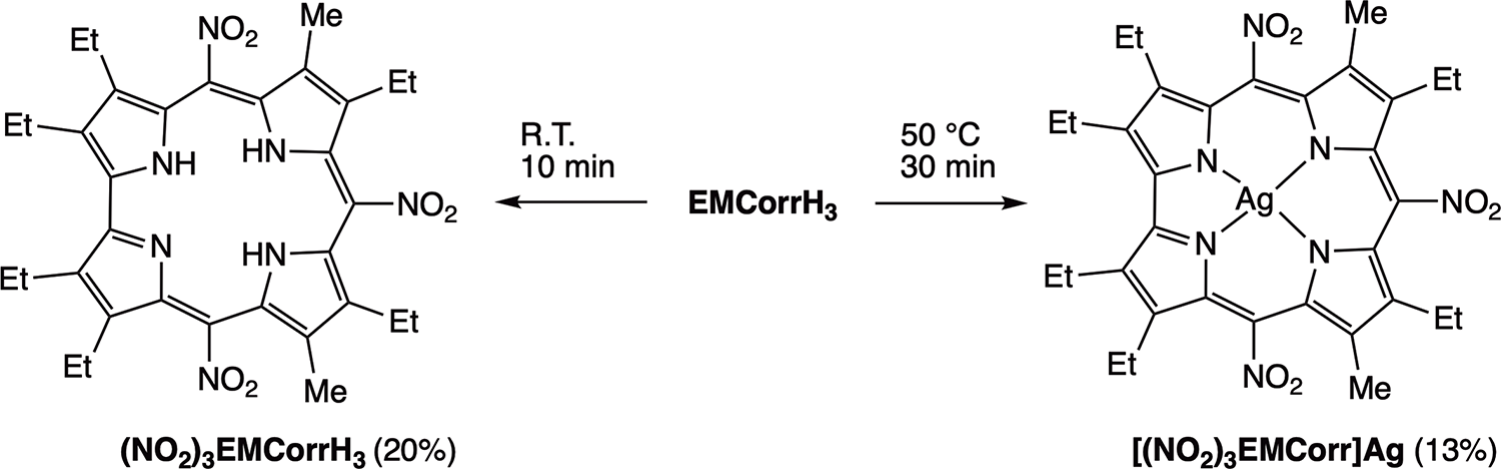
Products of the Reactions of EMCorrH_3_ (1 equiv) with AgNO_2_ (5 equiv) /NaNO_2_ (10 equiv) in CH_3_CN: Both Synthetic Pathways Led to the Formation of Oxocorroles as Byproducts

This compound was purified and fully characterized by UV–vis, ^1^H NMR, and mass spectrometry (Figures S13–S15).

In the ^1^H NMR spectrum, the lack of any resonance above 4 ppm indicates the complete substitution of the *meso* protons by the nitro groups. Furthermore, the formation of the nitrated silver complex is evidenced by the UV–vis spectrum; while maintaining an overall spectral profile similar to that of the parent Ag complex, it exhibits a broader Soret band and red-shifted Q-bands, suggesting an effective substitution with an electron-withdrawing group such as the nitro group.^21^ Its identification as the Ag­(III) corrolate fully substituted with −NO_2_ groups at the *meso-*positions was further confirmed by single-crystal X-ray crystallographic analysis (Figure 4).

**Figure 4:**
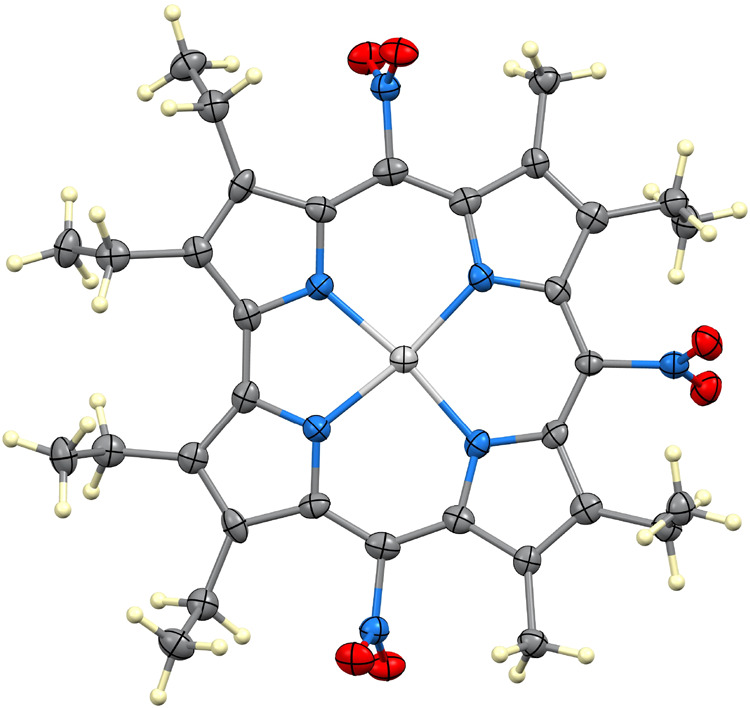
X-ray single-crystal structure of [(NO_2_)_3_EMCorr]­Ag. One of the two independent molecules is shown, with 50% ellipsoids.

The structure of [(NO_2_)_3_EMCorr]­Ag has two independent molecules, both lying on crystallographic 2-fold axes. The corrole cores have saddle conformations, with β carbon atoms an average of 0.298 Å alternately in pairs out of plane. The planes of the nitro groups form dihedral angles of average 74.3° with the corrole planes. The Ag–N distances to the directly bonded pyrroles are 1.945(4) and 1.957(4) Å, while those to the other pyrrole N atoms are longer, 1.970(4) and 1.979(4) Å.

Interestingly, when the reaction was carried out at room temperature and terminated after only 10 min, a green compound was obtained as the major product in a very good yield. Full spectroscopic characterization (Figures S13 and S16–S18) allowed its identification as (NO_2_)_3_EMCorrH_3_, confirming the high reactivity of the macrocycle toward *meso*-functionalization. As previously reported for aryl derivatives,^46^ nitrated compounds exhibit characteristic absorption profiles that feature multiple transitions within the Soret region and significantly red-shifted bands compared to the starting materials. Furthermore, their shape is strongly influenced by both the number of −NO_2_ groups introduced and their position on the macrocycle. This trend is also observed in the case of the alkylcorrole presented here. Specifically, the free-base trinitro derivative displays a Soret band shifted toward longer wavelengths by approximately 50 nm, while the multiple transitions corresponding to the Q-bands are replaced by a single, broad, and more intense absorption centered at 610 nm. Such an effect is ascribed to the synergistic combination of the strong electron-withdrawing character of the nitro groups and the macrocyclic distortion induced by steric hindrance with adjacent alkyl substituents.

These results support the reaction pathway proposed in Scheme 2.

Polynitration of the β-alkylcorrole at the *meso*-positions is proposed to occur via an oxidative nucleophilic aromatic substitution mechanism, which involves the formation of a π-cation radical intermediate. Upon treatment with silver nitrite, the free-base corrole undergoes single-electron oxidation by the Ag­(I) ion, generating a delocalized radical species that enhances the electrophilicity of the *meso* carbon atoms (C5, C10, and C15). This radical intermediate facilitates nucleophilic attack by nitrite ions, which are abundantly present in the reaction medium, thereby enabling sequential nitration at the *meso*-positions.^16^

The presence of β-alkyl substituents increases the electron density of the macrocycle and stabilizes the radical intermediate, promoting efficient polynitration without compromising the integrity of the macrocyclic framework. Subsequent metalation with silver­(I) salts occurs through the coordination of the Ag­(III) ion to the corrole core, whose electron density is significantly reduced by the electron-withdrawing nature of the −NO_2_ groups.

Finally, we focused on other silver salts with non-nucleophilic anions such as AgNO_3_ and AgPF_6_. This characteristic could be advantageous in preventing *meso*-functionalization in acetonitrile, potentially favoring the formation of silver corrole. Unfortunately, the use of these salts proved to be ineffective, resulting in complete decomposition of the starting material when AgNO_3_ was employed. In contrast, when AgPF_6_ was used, a small quantity of oxocorroles was recovered following the reaction workup. In both cases, neither functionalized products nor complexes with the silver ions were identified.

These results suggest that the presence of an electron-withdrawing group such as acetate or nitrite is crucial for stabilizing the (electron-rich) macrocycle in the presence of oxidant species, thereby limiting extensive corrole decomposition. At the same time, the reduced electron density, resulting from AcO and NO_2_ functionalization, facilitates the insertion of the silver ion. Accordingly, simultaneous corrole functionalization and metalation were observed in the presence of AgOAc and AgNO_2_, whereas both AgNO_3_ and AgPF_6_ were ineffective.

## Conclusions

Corrole has once again proven to exhibit less predictable and less straightforward reactivity compared to the more extensively studied porphyrin macrocycle. The synthetic strategy that readily afforded silver complexes of *meso*-triarylcorroles in satisfactory yields failed for β-alkylcorroles, yielding only trace amounts of the desired complex.

The reaction was found to be highly sensitive to both the choice of the solvent and the silver source. The use of AgOAc in pyridine afforded oxocorroles arising from macrocycle oxidation as the main products, whereas the same reaction in acetonitrile afforded a broader product distribution. In addition to oxocorroles, *meso*-functionalization by the acetate moiety was observed. This modification markedly alters macrocycle reactivity, enabling either subsequent metalation ([(AcO)­EMCorr]Ag and [(AcO)_2_EMCorr]­Ag) or further oxidation to yield a functionalized oxocorrole (15-AcO-5-OxoCorrH_2_), not previously reported.

Building on these findings, replacement of acetate with nitrite revealed that simple modulation of the reaction time enables corrole polynitration, giving the free base ((NO_2_)_3_EMCorrH_3_) and the silver complex ([(NO_2_)_3_EMCorr]­Ag). As a confirmation, silver salts bearing non-nucleophilic anions led exclusively to starting material decomposition/oxidation.

Oxocorroles, readily formed via macrocycle oxidation, are of particular interest as rare examples of antiaromatic compounds, as indicated by the unusual chemical shifts of the *meso*- and inner-core protons. To date, only a limited number of studies have addressed antiaromatic porphyrinoids;^32,48^ taking advantage of their relatively straightforward formation from alkylcorroles, we aim to further investigate this intriguing class of macrocycles.

## Supplementary Material



## Notes

Chemical structure of the substrate EMCorrH_3_, ^1^H NMR, UV–vis, and mass spectra (PDF)

## Abbreviations

TLC: thin-layer chromatography
NMR: nuclear magnetic resonance
UV–vis: ultraviolet–visible
AP-MALDI-HRMS: atmospheric pressure matrix-assisted laser desorption/ionization high-resolution mass spectrometry.
