# Supplementary material for: Functionalization of β‑Alkylcorrole Using Ag(I) Salts
Source: Inorg Chem. 2026 Jul 27;65(31):18064–73. doi: 10.1021/acs.inorgchem.6c02241 (PMC13463540; doi:10.1021/acs.inorgchem.6c02241)
Supplement: Supplementary file 1 [file ic6c02241_si_001.pdf]

Supporting Information

Functionalization of β-Alkylcorrole Using Ag(I) Salts

Martina Marsotto[1], Giuseppe Pomarico[2,], Manuela Stefanelli[1], Anna Troiani[3], Frank R. Fronczek[4], Kevin M. Smith[4], Roberto Paolesse[1]*

AUTHOR ADDRESS

[1] Department of Chemical Science and Technologies, University of Rome Tor Vergata, Rome 00133, Italy

[2] Department of Chemistry, Sapienza - University of Rome, Rome 00185, Italy; Email: giuseppe.pomarico@uniroma1.it

[3] Department of Chemistry and Technology of Drugs, Sapienza - University of Rome, Rome 00185, Italy;

[4] Department of Chemistry, Louisiana State University, Baton Rouge, Louisiana 70803, United States

Table of contents

HRMS experimental details S3
Figure S1. Chemical structure of $EMCorrH_3$ S4
Figure S2. UV-Vis spectra of Ag-corrolates S4
Figure S3. AP-MALDI-HRMS, positive ion mode of [EMCorr]Ag S5
Figure S4. UV-Vis spectra of oxocorroles S6
Figure S5. ^{1}H NMR spectrum of 5-$OxoCorrH_2$ S7
Figure S6. ^{1}H NMR spectrum of 10-$OxoCorrH_2$ S7
Figure S7. ESI mass spectrum of 5-$OxoCorrH_2$ S8
Figure S8. ESI mass spectrum of 10-$OxoCorrH_2$ S8
Figure S9. AP-MALDI-HRMS, positive ion mode of [(AcO)EMCorr]Ag S9
Figure S10. ^{1}H NMR spectrum of 15-AcO-5-$OxoCorrH_2$ S10
Figure S11. AP-MALDI-HRMS, positive ion of 15-AcO-5-$OxoCorrH_2$ S11
Figure S12. AP-MALDI-HRMS, positive ion mode $[(AcO)_2EMCorr]Ag$ S12
Figure S13. UV-Vis spectra of $(NO_2)_3EMCorr$ and $[(NO_2)_3EMCorr]Ag$ S13
Figure S14. ^{1}H NMR spectrum of $[(NO_2)_3EMCorr]Ag$ S13
Figure S15. AP-MALDI-HRMS, negative ion mode $[(NO_2)_3EMCorr]Ag$ S14
Figure S16. ^{1}H NMR spectrum of $(NO_2)_3EMCorr$ S15
Figure S17. AP-MALDI-HRMS, positive ion mode $(NO_2)_3EMCorr$ S16
Figure S18. AP-MALDI-HRMS, negative ion mode $(NO_2)_3EMCorr$ S17

High-Resolution Mass Spectrometry Measurements (AP-MALDI-HRMS)

Accurate mass measurements were performed using an Orbitrap Exploris 120 mass spectrometer (ThermoFisher Scientific, Waltham, MA, USA) coupled to an atmospheric pressure matrix-assisted laser desorption/ionization (AP-MALDI) ion source (next generation ultra-high resolution ion source, ng-UHR; MassTech Inc., Columbia, MD, USA). Approximately 1 mg of each synthesized compound was added to 200 μL of acetonitrile and sonicated for several minutes in an ultrasonic bath. A 1 μl aliquot of the resulting suspension was spotted onto the wells of the MALDI target plate. For positive-ion mode acquisition, each spot was overlaid with an equal volume of a 5 mg mL^{-1} solution of alpha-cyano-4-hydroxycinnamic acid (CHCA, LaserBio Labs, France) prepared in acetonitrile/ultrapure water/trifluoroacetic acid (50:50:0.1 v/v/v). For both positive- and negative-ion mode acquisition, 1,2-diamino naphthalene (DAN; Merck) was used as the matrix, prepared as a 5mg mL^{-1} solution in acetonitrile/ultrapure water (50:50 v/v). After solvent evaporation under ambient conditions, the co-crystallized samples were analyzed.

Samples were irradiated with a UV laser (λ = 355 nm) operating in spiral motion mode at a repetition rate of 1000 Hz and 1% laser energy. Additional source parameters, including ionization voltage and ion transfer tube temperature, were optimized on a per-compound basis to maximize signal intensity. Mass spectra were acquired in both positive- and negative ion modes over the *m/z* range of 400-1000 Da with a resolving power of 120000 at *m/z* 200 and an injection time of 100 ms. The instrument was calibrated daily using the Flexmix Calibration solution (ThermoFisher Scientific). Scan-to-scan mass scale recalibration was performed using fluorantrene as the internal lock mass, introduced via the EASY-IC source option. Raw data were processed with FreeStyle software v. 1.8 (ThermoFisher Scientific, included in the Xcalibur package). Mass accuracy below 2 ppm was achieved between theoretical and experimental *m/z* values for all detected species. Depending on the electronic and chemical properties of each compound, ionized species were detected as protonated $[M + H]^{+}$ or deprotonated $[M - H]^{-}$ molecules, or radical cations/anions $[M]^{+\bullet}$/$[M]^{-\bullet}$, as specified for each compound in the relevant section.

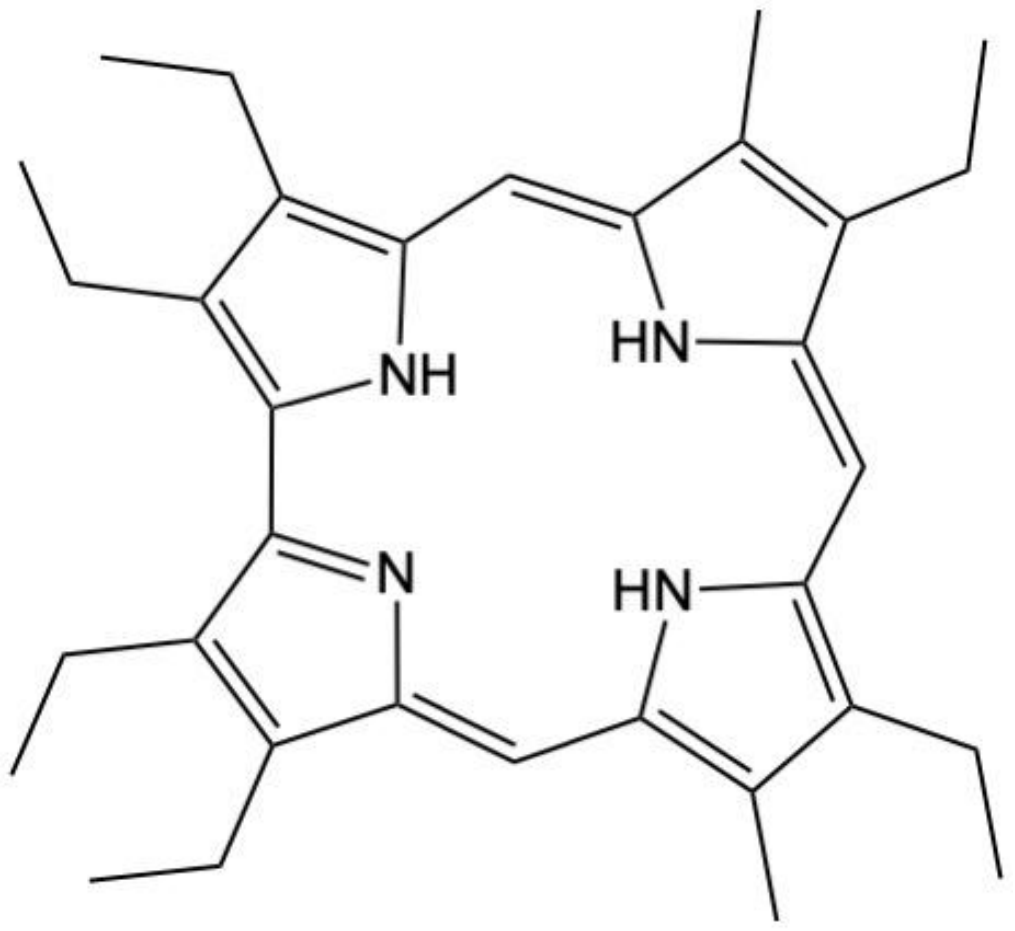

Figure S1. Chemical structure of $EMCorrH_3$.

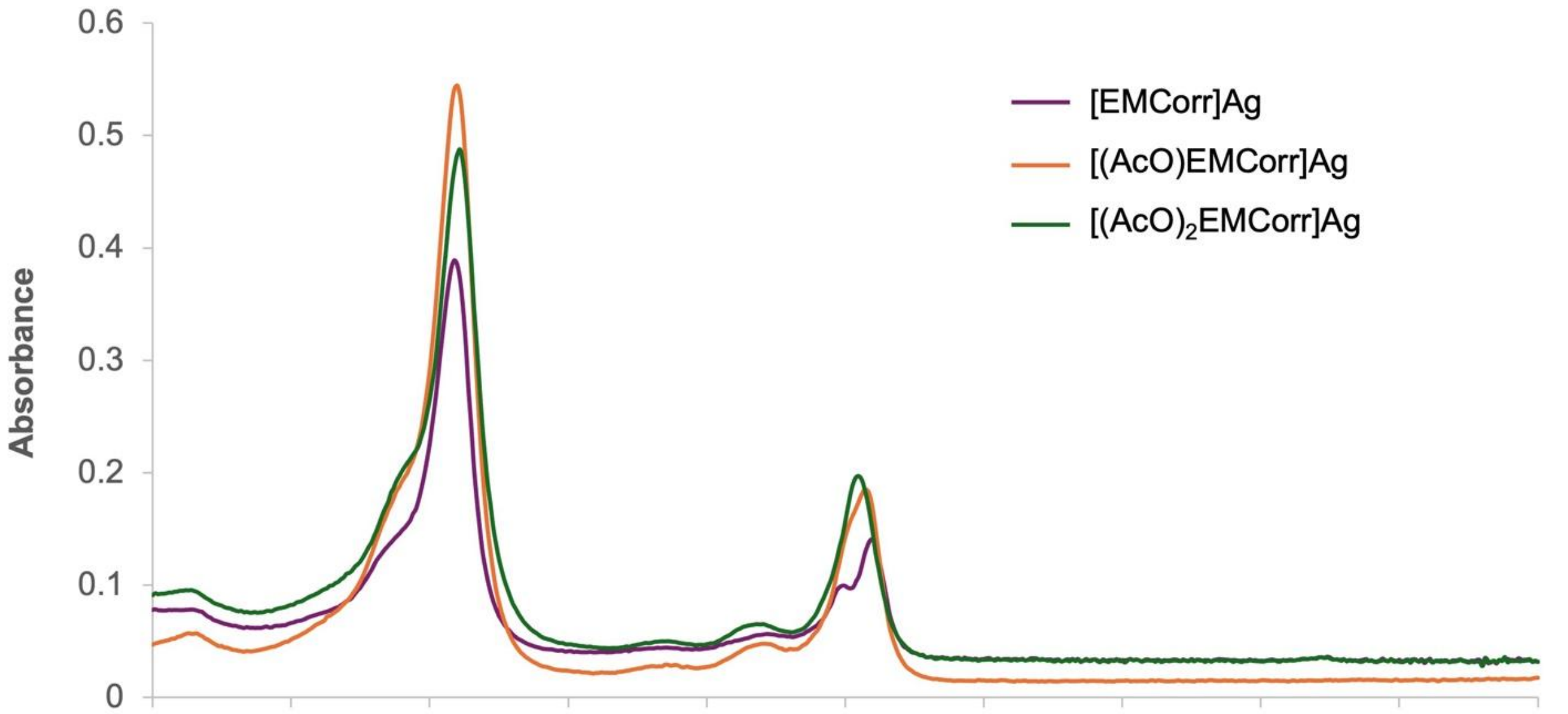

Figure S2. UV-Vis spectra of Ag-corrolates in CH_2Cl_2.

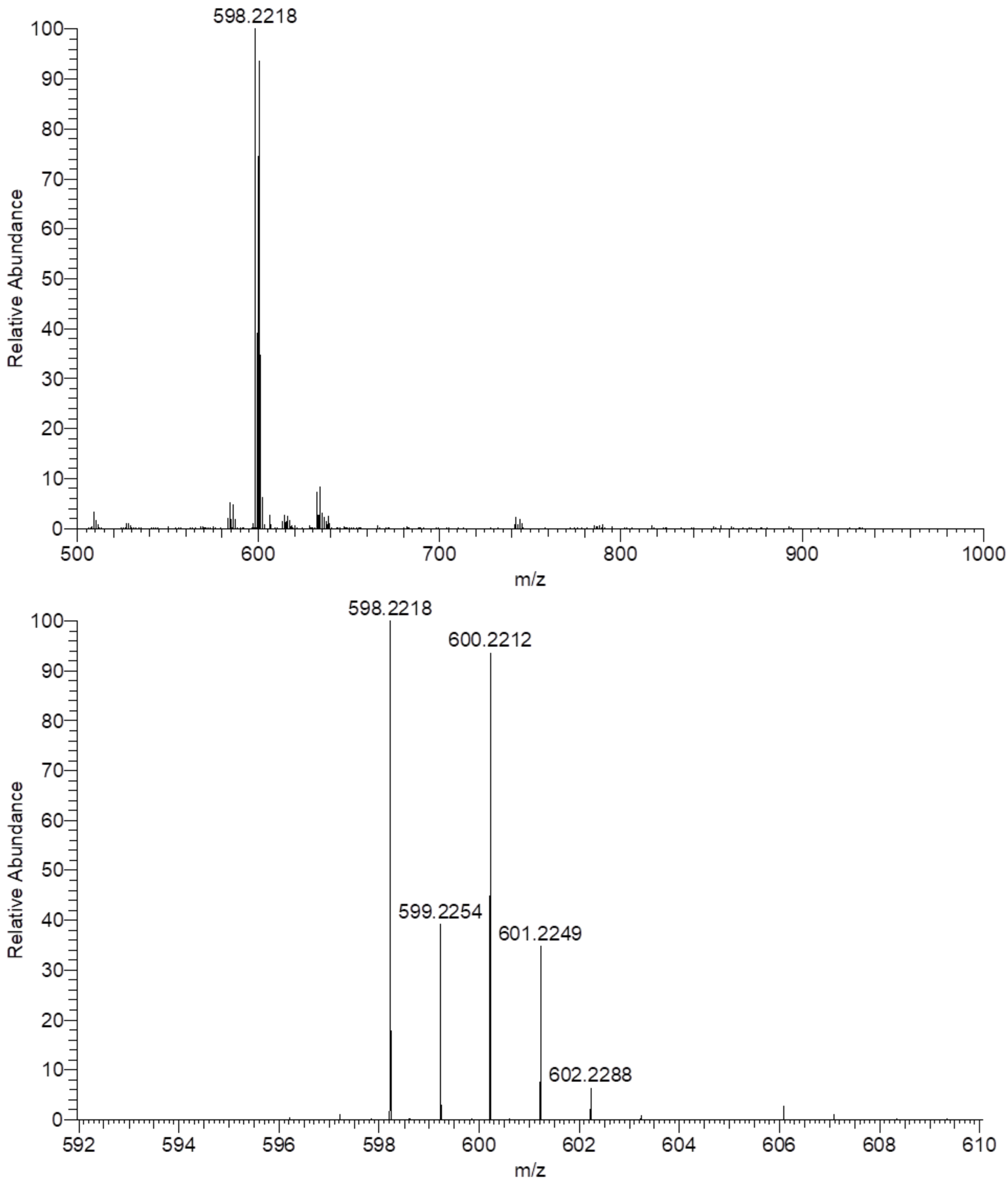

Figure S3. AP-MALDI-HRMS positive ion mode mass spectrum of [EMCorr]Ag in the 500-1000 *m/z* range using DAN as matrix and expansion of the 592-610 *m/z* region showing the isotope pattern and signal assignment for the radical cation $[C_{33}H_{39}AgN_4]^{+\bullet}$: *m/z* 598.2218, (calcd 598.2220 for $[C_{33}H_{39}{}^{107}AgN_4]^{+\bullet}$, error: -0.33 ppm) and *m/z*: 600.2212 (calcd for $[C_{33}H_{39}{}^{109}AgN_4]^{+\bullet}$ *m/z*: 600.2217 error: -0.83 ppm).

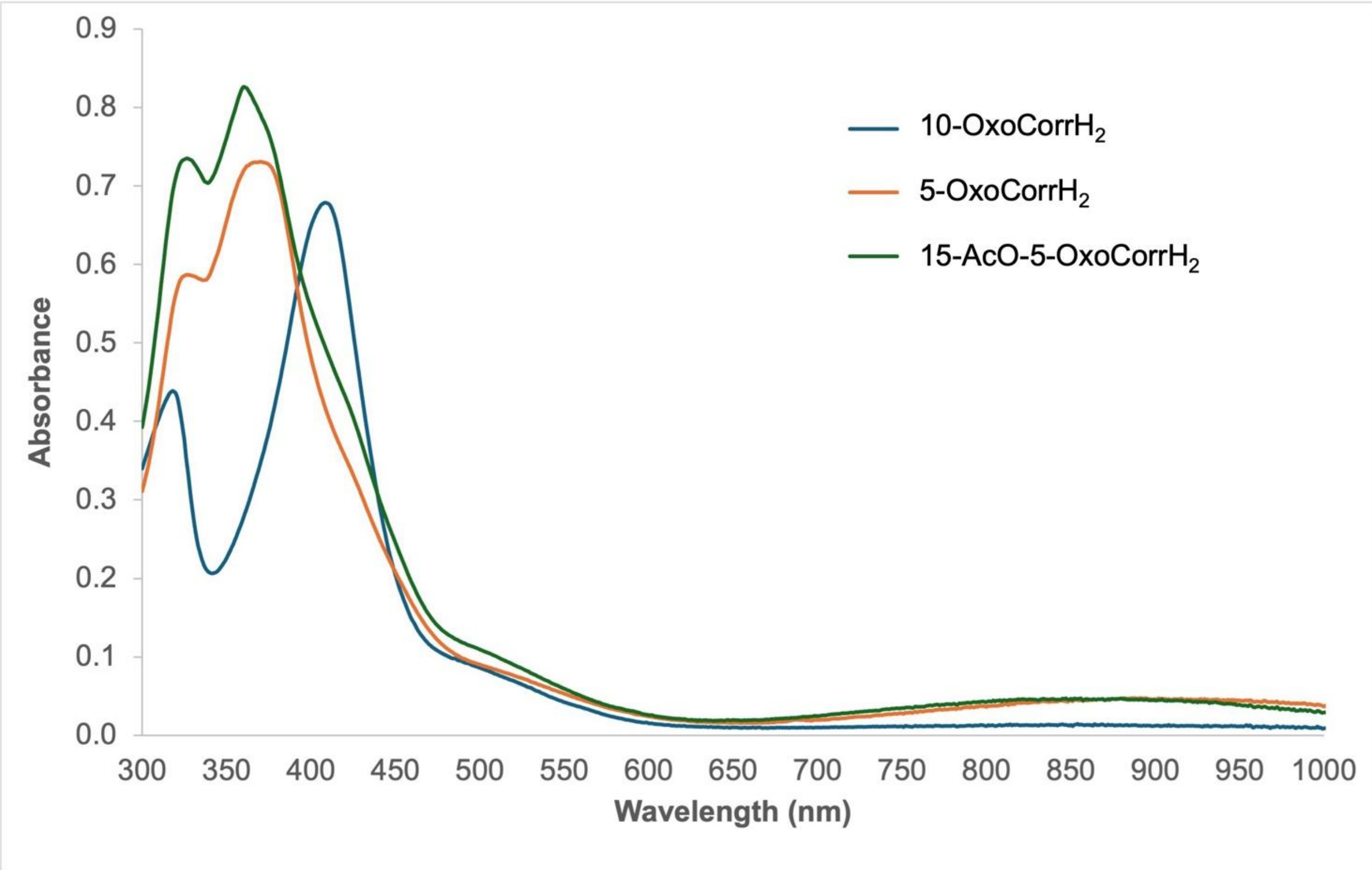

Figure S4. UV-Vis spectra of 5-OxoCorrH_2, 10-OxoCorrH_2, and 15-AcO-5-OxoCorrH_2 in CH_2Cl_2.

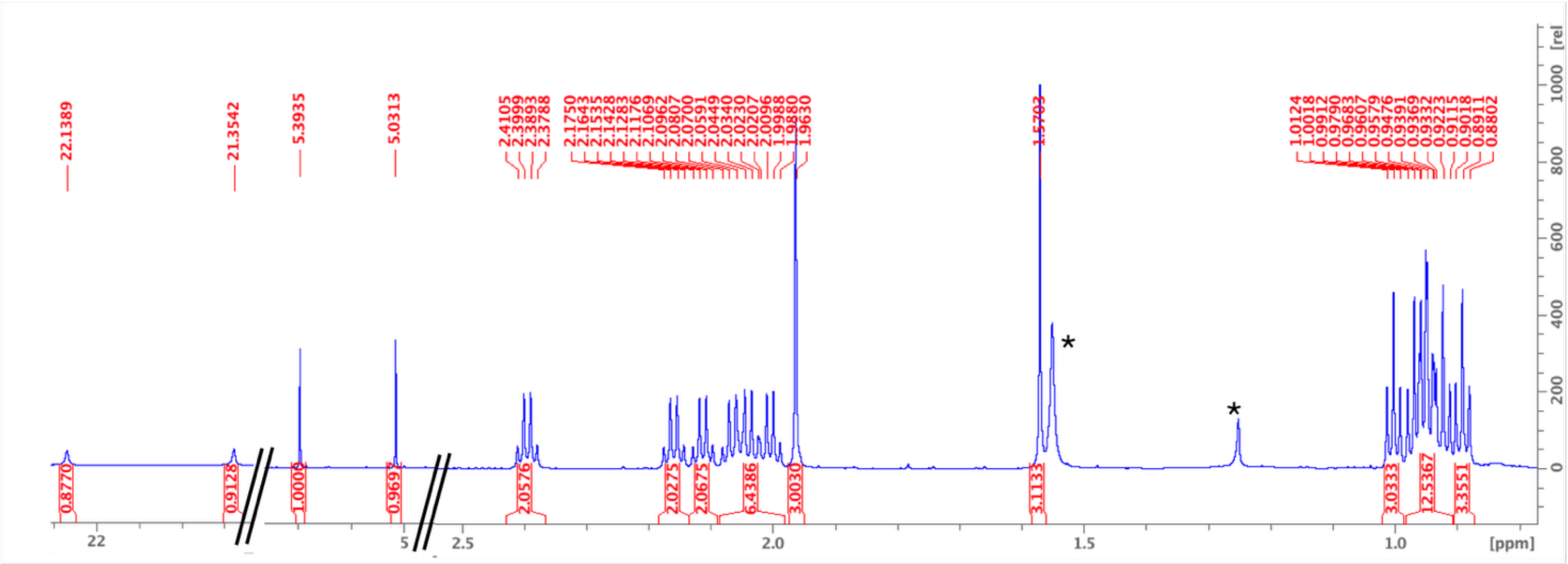

Figure S5. ^{1}H NMR spectrum of 5-OxoCorrH_2 in $CDCl_3$. Peaks marked with an asterisk correspond to residual solvent or impurities.

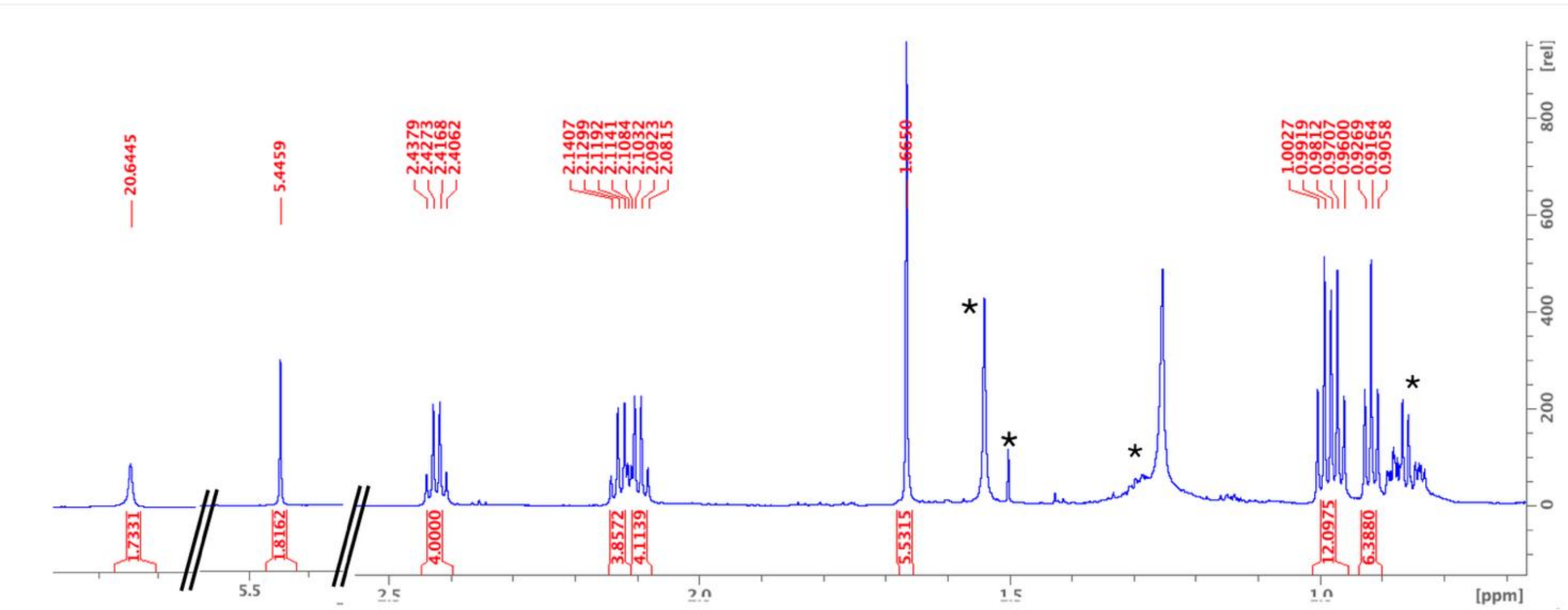

Figure S6. ^{1}H NMR spectrum of 10-OxoCorrH_2 in $CDCl_3$. Peaks marked with an asterisk correspond to residual solvent or impurities.

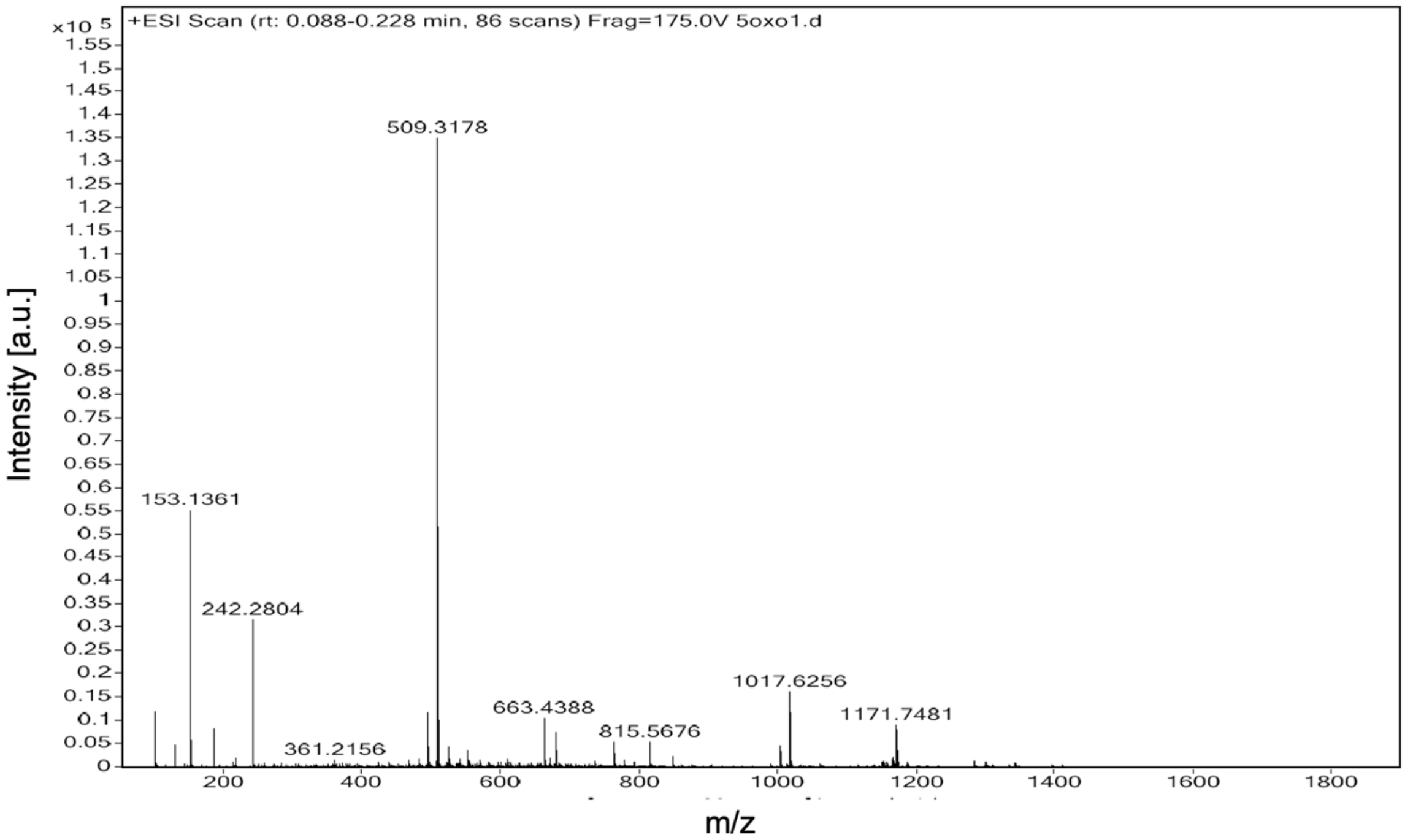

Figure S7. ESI mass spectrum of 5-OxoCorrH_2.

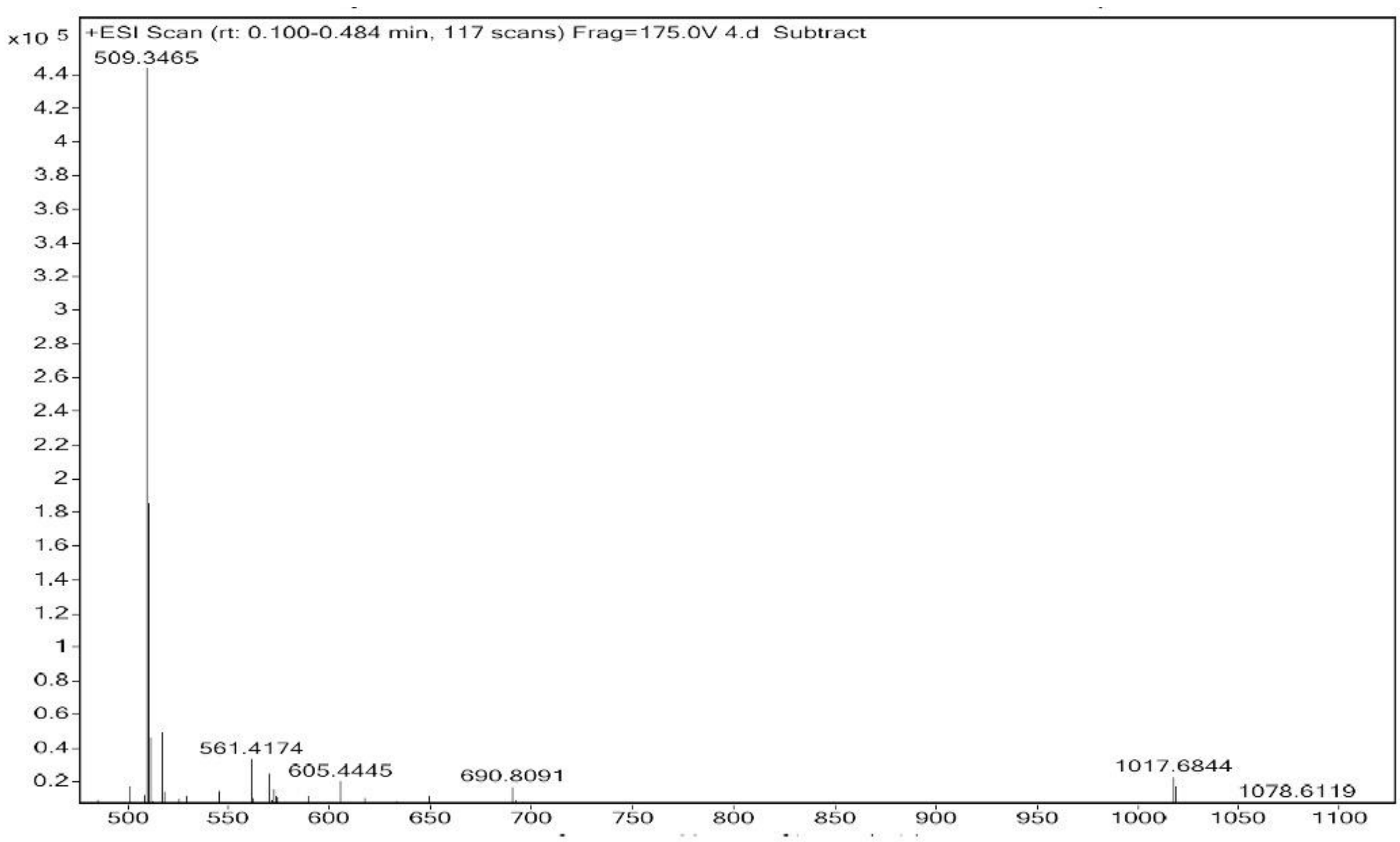

Figure S8. ESI mass spectrum of 10-OxoCorrH_2.

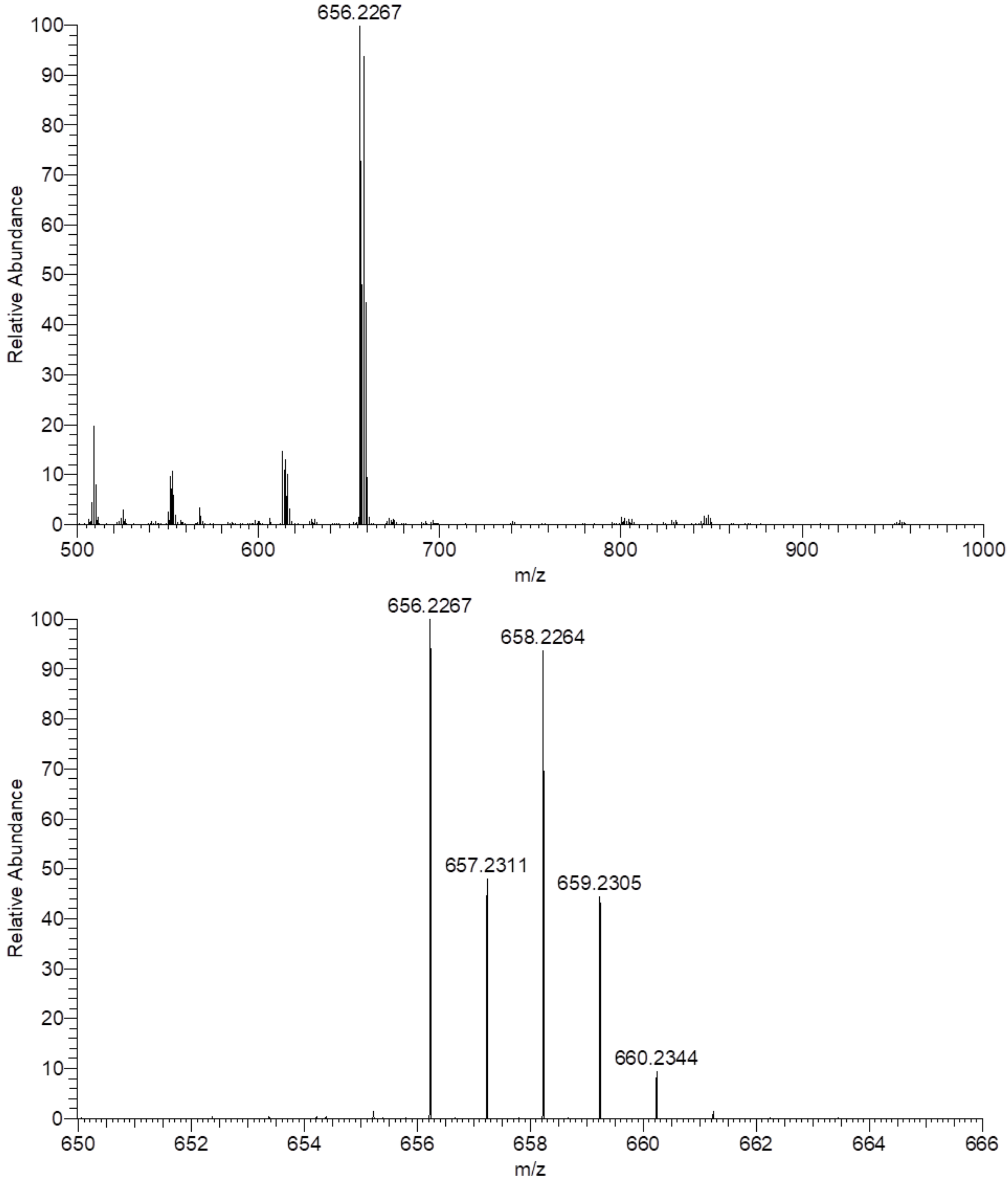

Figure S9. AP-MALDI-HRMS positive ion mode mass spectrum of [(AcO)EMCorr]Ag in the 500-1000 *m/z* range using DAN as matrix and expansion of the 650-666 *m/z* region showing the isotope pattern and signal assignment for the radical cation $[C_{35}H_{41}AgN_4O_2]^{+\bullet}$: *m/z* 656.2267 (calcd for $[C_{35}H_{41}{}^{107}AgN_4O_2]^{+\bullet}$ *m/z* 656.2275, error: -1.20 ppm) and *m/z*: 658.2264 (calcd for $[C_{35}H_{41}{}^{109}AgN_4O_2]^{+\bullet}$ *m/z* 658.2272, error: -1.21 ppm).

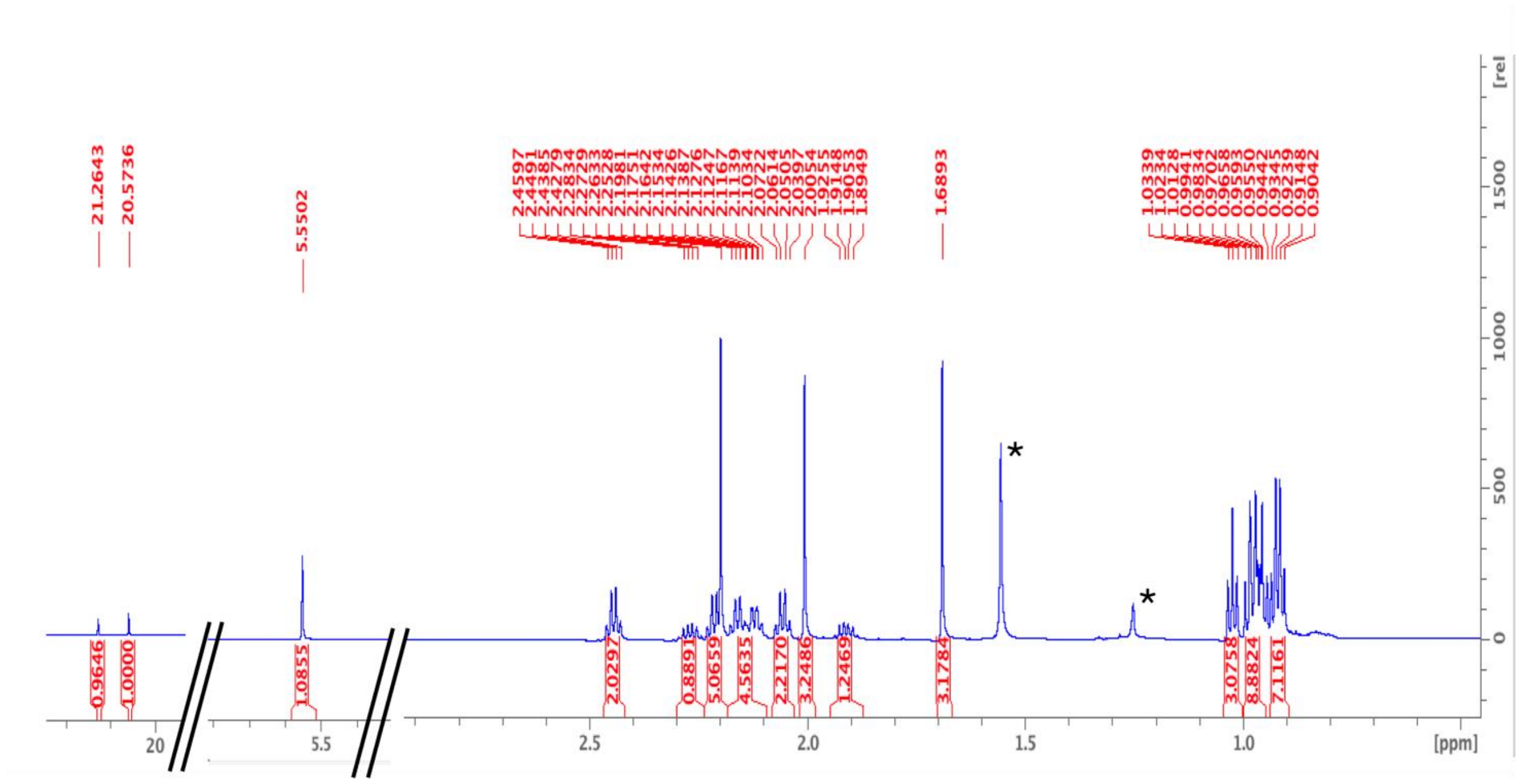

Figure S10. ^1H NMR spectrum of 15-AcO-5-OxoCorrH_2 in $CDCl_3$. Peaks marked with an asterisk correspond to residual solvent or impurities.

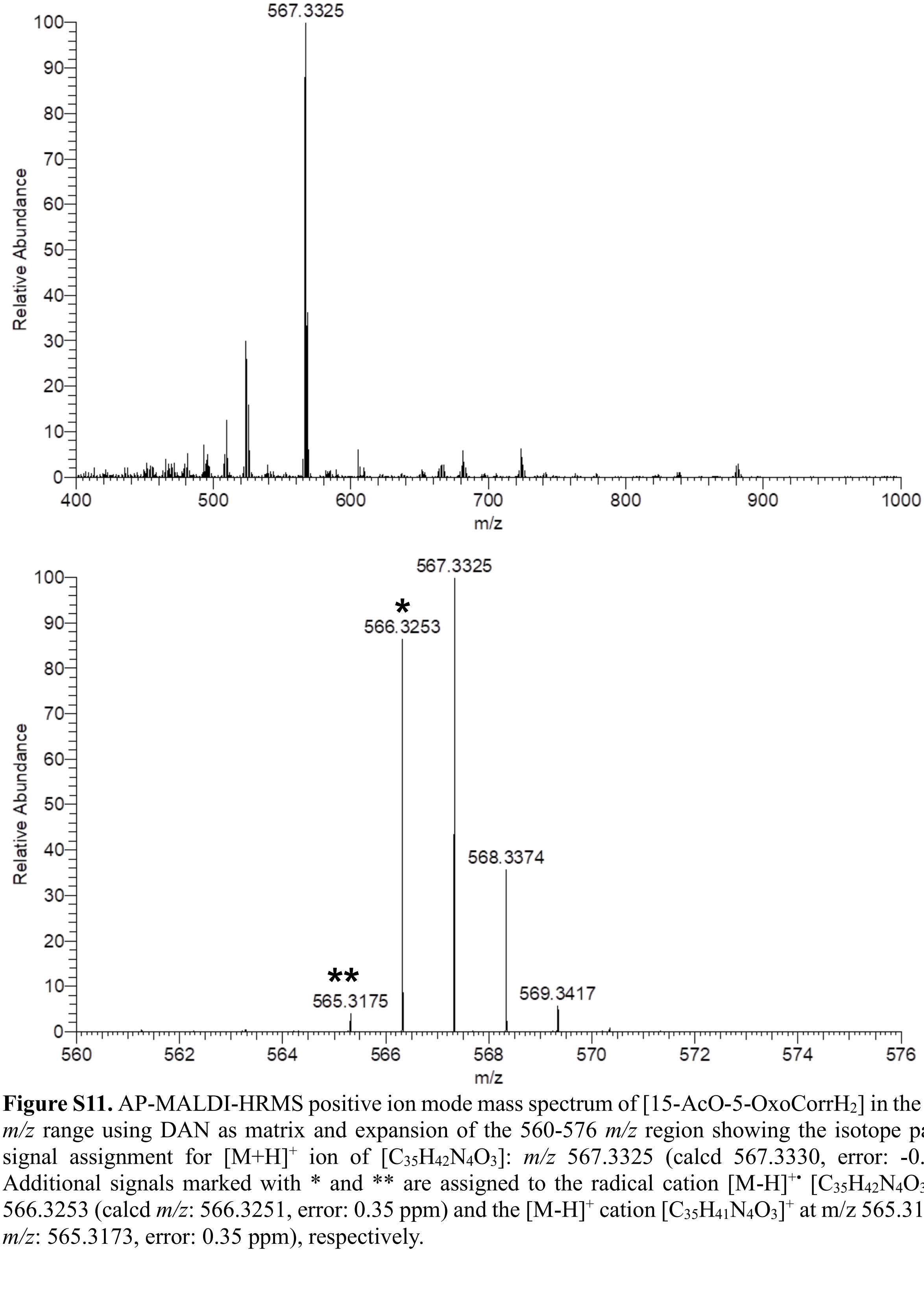

Figure S11. AP-MALDI-HRMS positive ion mode mass spectrum of [15-AcO-5-OxoCorrH_2] in the 400-1000 *m/z* range using DAN as matrix and expansion of the 560-576 *m/z* region showing the isotope pattern and signal assignment for $[M+H]^+$ ion of [$C_{35}H_{42}N_4O_3$]: *m/z* 567.3325 (calcd 567.3330, error: -0.88 ppm). Additional signals marked with * and ** are assigned to the radical cation $[M-H]^{+\bullet}$ $[C_{35}H_{42}N_4O_3]^{+\bullet}$ at *m/z* 566.3253 (calcd *m/z*: 566.3251, error: 0.35 ppm) and the $[M-H]^+$ cation $[C_{35}H_{41}N_4O_3]^+$ at m/z 565.3175 (calcd *m/z*: 565.3173, error: 0.35 ppm), respectively.

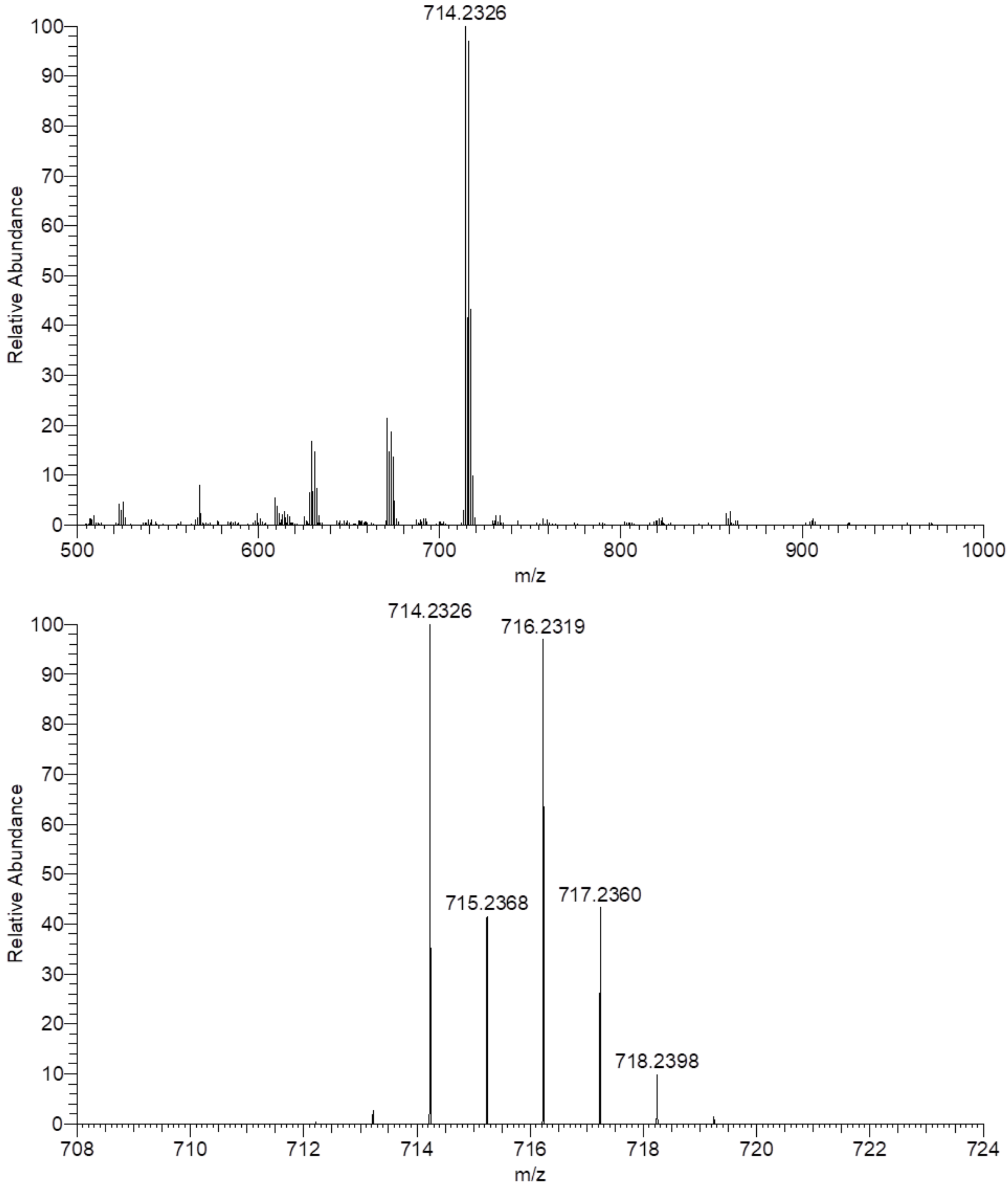

Figure S12. AP-MALDI-HRMS positive ion mode mass spectrum of [(AcO)$_2$EMCorr]Ag in the 500-1000 *m/z* range using CHCA as matrix and expansion of the 708-724 *m/z* region showing the isotope pattern and signal assignment for the radical cation [$C_{37}H_{43}AgN_4O_4$]$^{+\bullet}$: *m/z* 714.2326 (calcd for [$C_{37}H_{43}{}^{107}AgN_4O_2$]$^{+\bullet}$ *m/z* 714.2330, error: -0.56 ppm) and *m/z* 716.2319 (calcd *m/z* 716.2326, for ([$C_{37}H_{43}{}^{109}AgN_4O_4$]$^{+\bullet}$ error: -0.98 ppm).

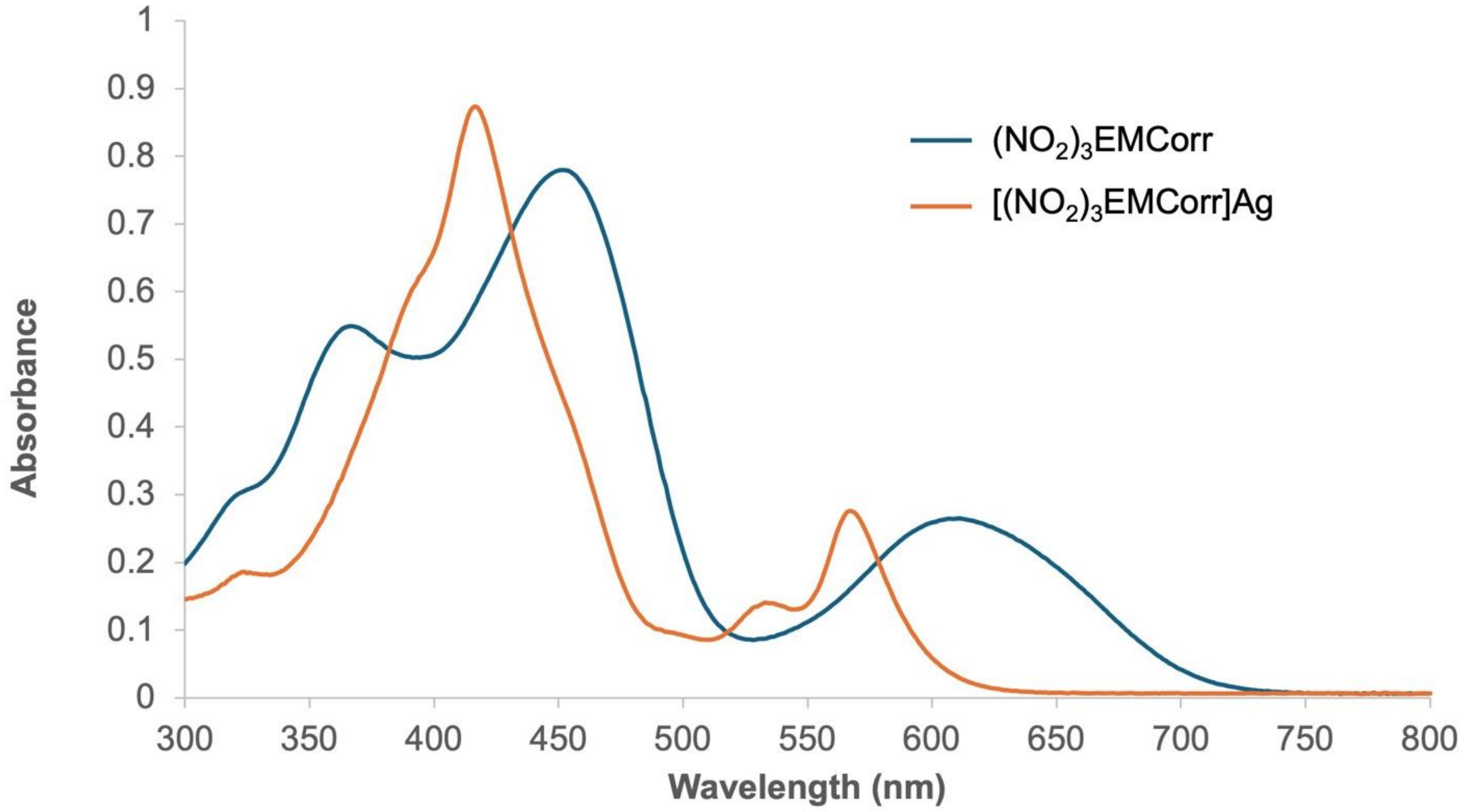

Figure S13. UV-Vis spectra of $(NO_2)_3$EMCorr and $[(NO_2)_3$EMCorr]Ag in CH_2Cl_2

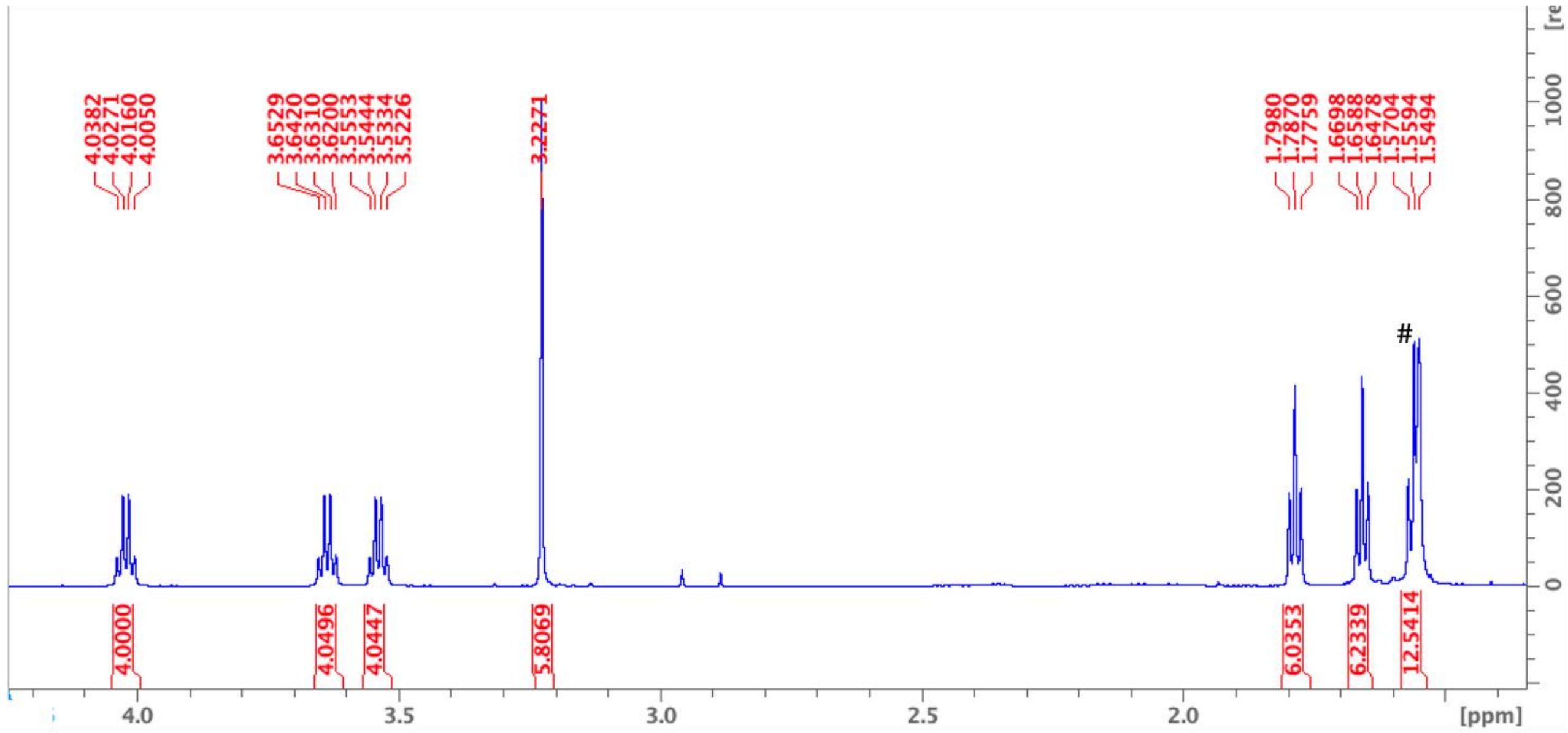

Figure S14. ^{1}H NMR spectrum of $[(NO_2)_3$EMCorr]Ag in $CDCl_3$; (#) denotes signal of residual water (1.55 ppm) overlapped to $-CH_3$ peak.

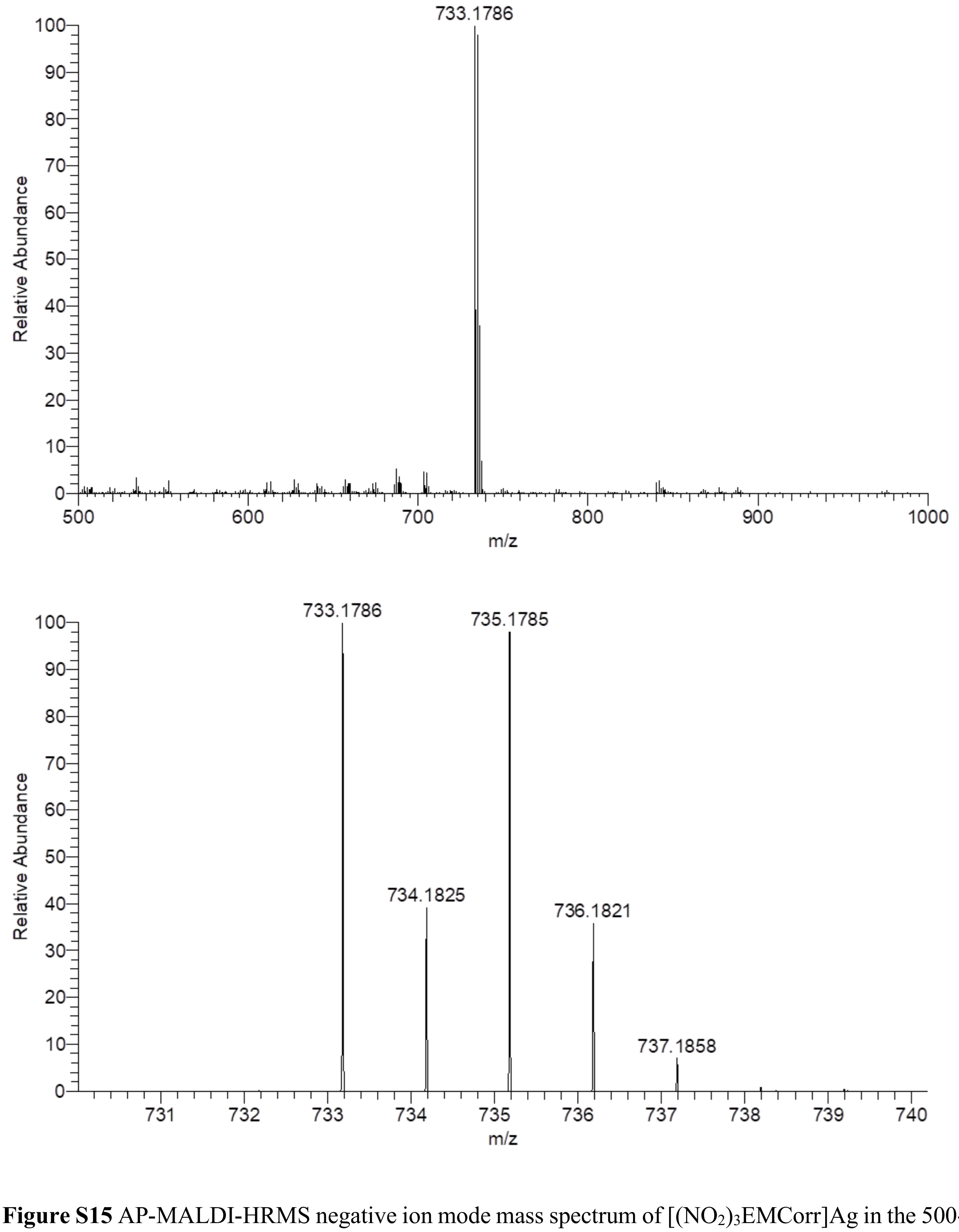

Figure S15 AP-MALDI-HRMS negative ion mode mass spectrum of $[(NO_2)_3EMCorr]Ag$ in the 500-1000 *m/z* range using DAN as matrix and expansion of the 730-740 *m/z* region showing the isotope pattern and signal assignment for the radical anion $[C_{33}H_{36}AgN_7O_6]^{-\bullet}$: *m/z* 733.1786 (calcd for $[C_{33}H_{36}{}^{107}AgN_7O_6]^{\cdot-}$ *m/z* 733.1784, error: 0.27 ppm) and *m/z* 735.1785 (calcd for ($[C_{33}H_{36}{}^{109}AgN_7O_6]^{\cdot-}$ *m/z* 735.1780, error: 0.68 ppm)

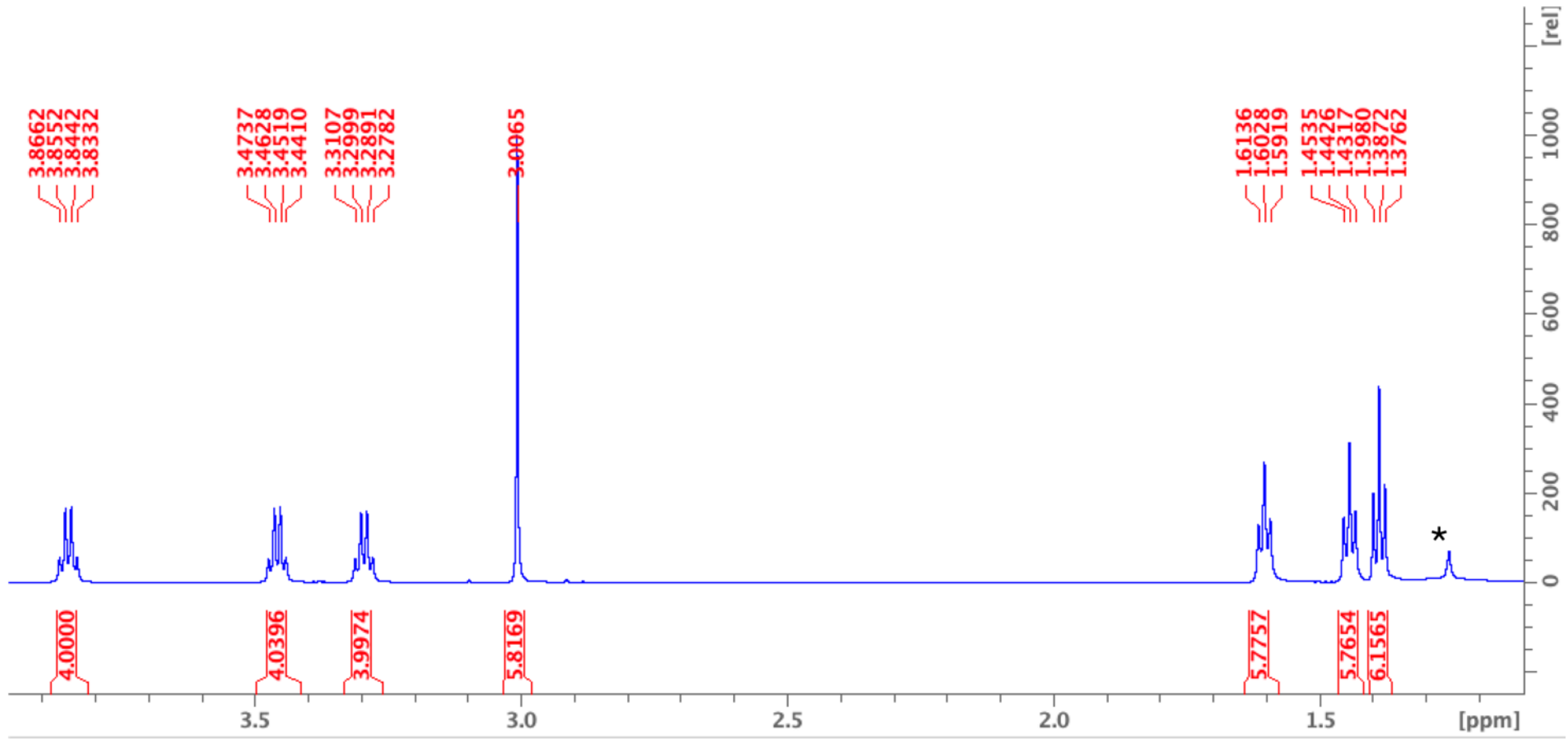

Figure S16. ^{1}H NMR spectrum of $(NO_2)_3$EMCorr in $CDCl_3$. Peaks marked with an asterisk correspond to residual solvent or impurities.

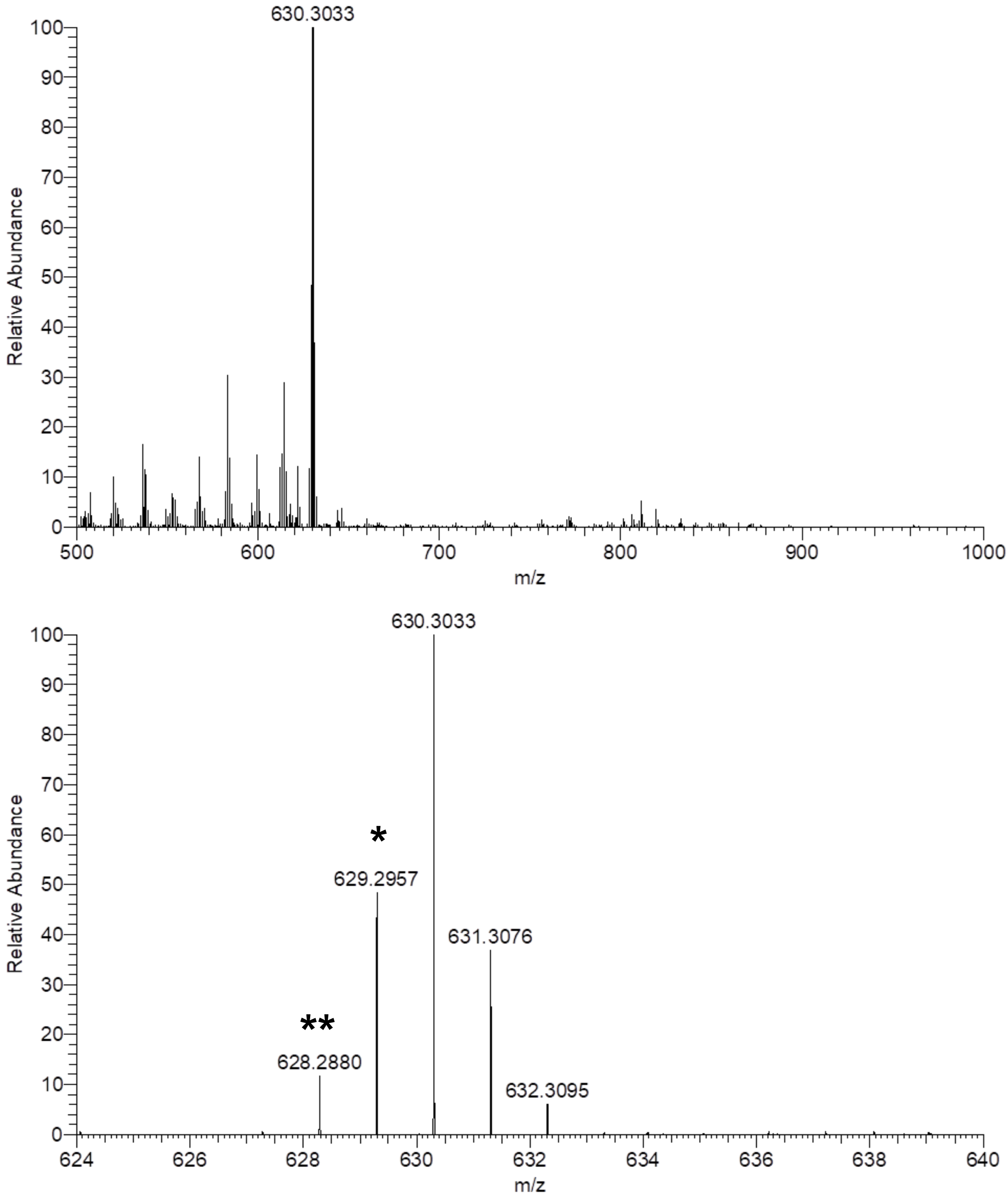

A) A)

Figure S17. AP-MALDI-HRMS positive ion mode mass spectrum of $(NO_2)_3$EMCorr in the 500-1000 *m/z* range using CHCA as matrix and expansion of the 624-640 *m/z* region showing the isotope pattern and signal assignment for the $[M+H]^+$ ion of $[C_{33}H_{39}N_7O_6]$: *m/z* 630.3033, (calcd *m/z*: 630.3035, error: -0.32 ppm). Additional signals marked with * and ** are assigned to the radical cation $[M]^{+\bullet}$ $[C_{33}H_{39}N_7O_6]^{+\bullet}$ at *m/z* 629.2957 (calcd 629.2956, error 0.16 ppm) and the $[M-H]^+$ cation $[C_{33}H_{38}N_7O_6]^+$ at *m/z* 628.2880 (calcd 628.2878, error 0.32 ppm), respectively.

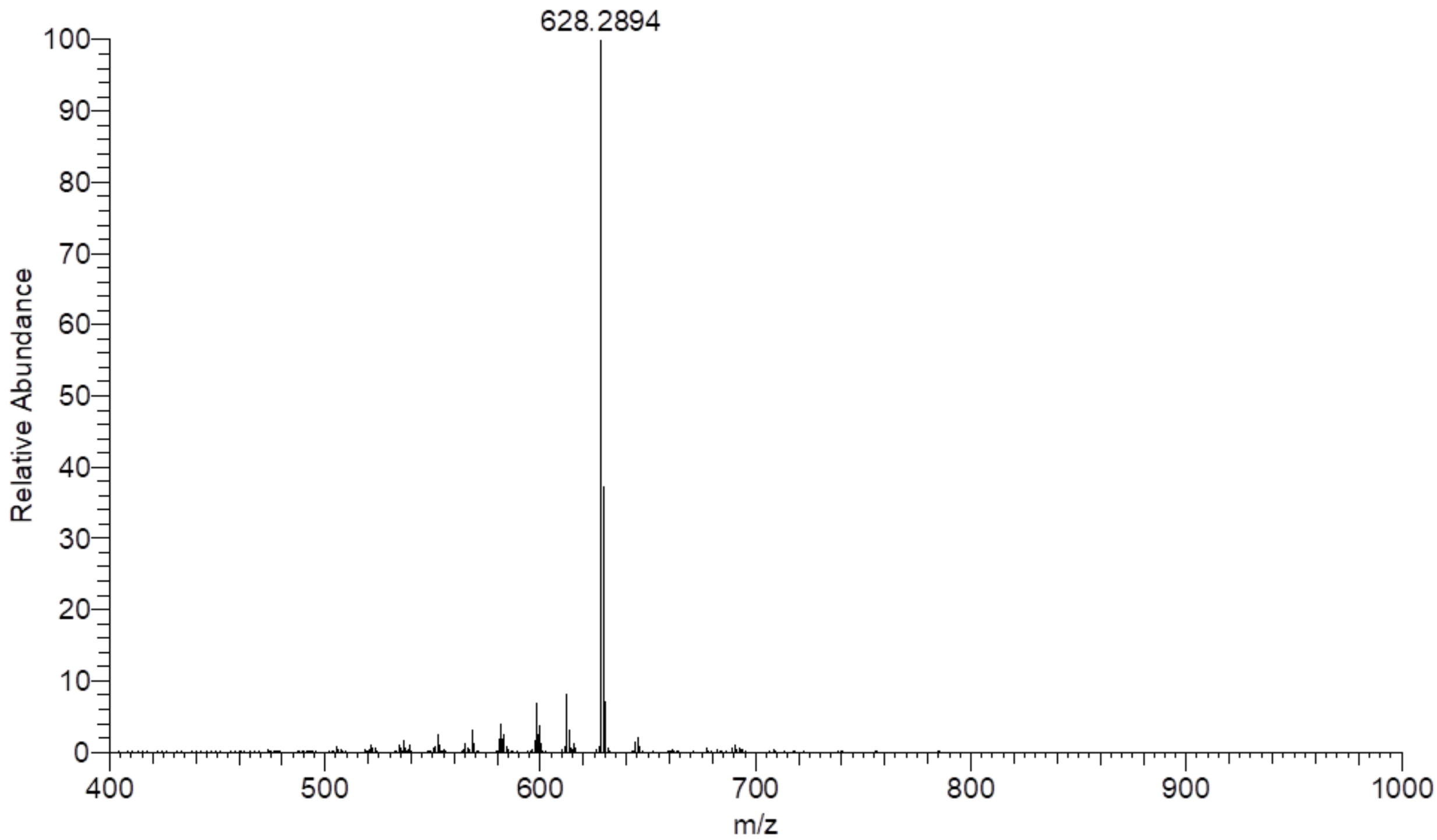

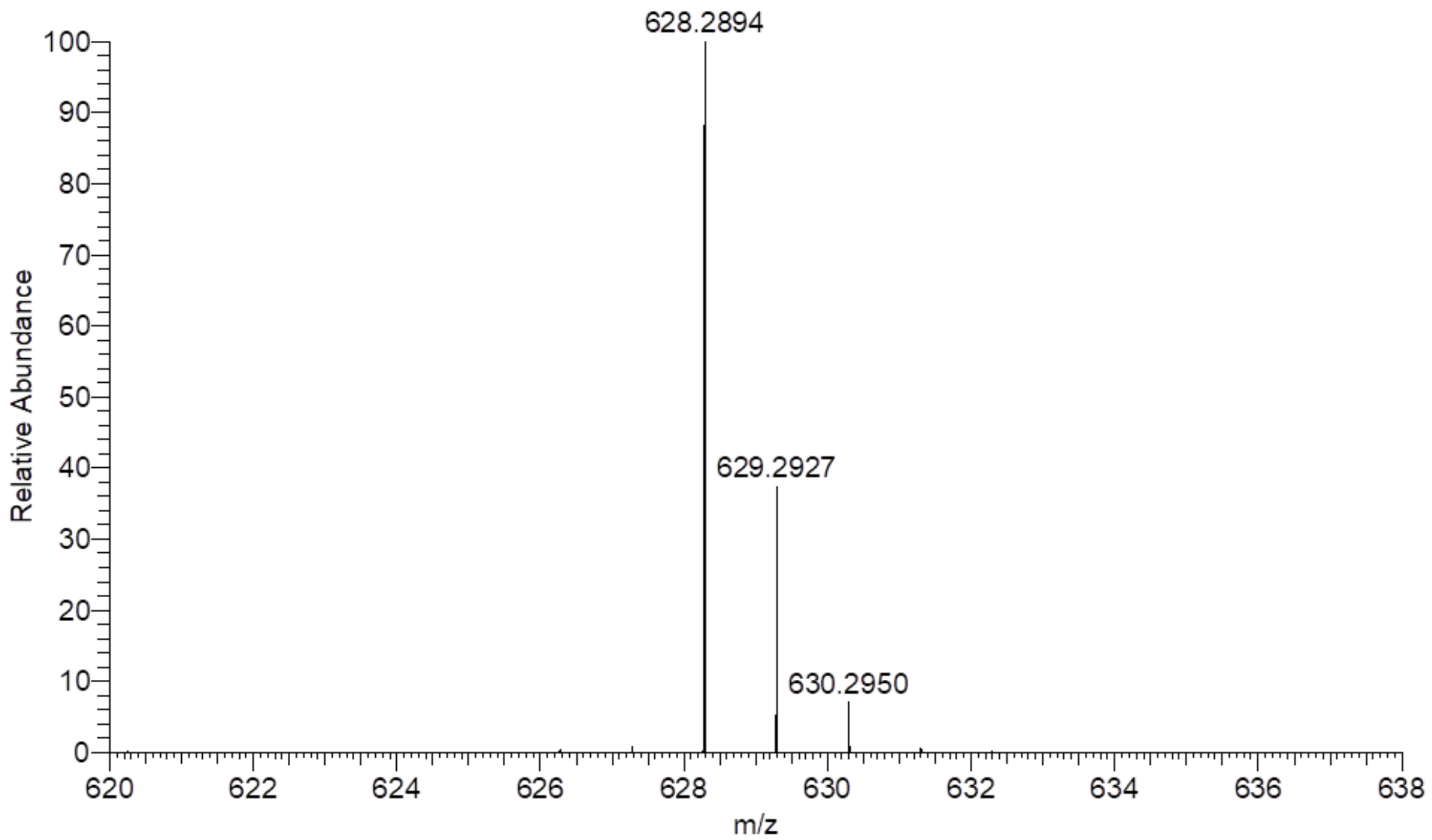

Figure S18. AP-MALDI-HRMS negative ion mode mass spectrum of $(NO_2)_3$EMCorr in the 400-1000 *m/z* range using DAN as matrix and expansion of the 620-638 *m/z* region showing the isotope pattern and signal assignment for the $[M\text{-}H]^-$ ion of $[C_{33}H_{39}N_7O_6]$: *m/z* 628.2894 (calcd 628.2889, error: 0.80 ppm).
